# Efficient syntheses of climate relevant isoprene nitrates and (1*R*,5*S*)-(−)-myrtenol nitrate

**DOI:** 10.3762/bjoc.12.103

**Published:** 2016-05-27

**Authors:** Sean P Bew, Glyn D Hiatt-Gipson, Graham P Mills, Claire E Reeves

**Affiliations:** 1 School of Chemistry, University of East Anglia, Norwich Research Park, Norwich, NR4 7TJ, UK; 2 Centre for Ocean and Atmospheric Science, School of Environmental Sciences, University of East Anglia, Norwich Research Park, Norwich, NR4 7TJ, UK

**Keywords:** atmospheric chemistry, exchange reaction, halide, isoprene nitrate, monoterpene

## Abstract

Here we report the chemoselective synthesis of several important, climate relevant isoprene nitrates using silver nitrate to mediate a ’halide for nitrate’ substitution. Employing readily available starting materials, reagents and Horner–Wadsworth–Emmons chemistry the synthesis of easily separable, synthetically versatile ‘key building blocks’ (*E*)- and (*Z*)-3-methyl-4-chlorobut-2-en-1-ol as well as (*E*)- and (*Z*)-1-((2-methyl-4-bromobut-2-enyloxy)methyl)-4-methoxybenzene has been achieved using cheap, ’off the shelf’ materials. Exploiting their reactivity we have studied their ability to undergo an ‘allylic halide for allylic nitrate’ substitution reaction which we demonstrate generates (*E*)- and (*Z*)-3-methyl-4-hydroxybut-2-enyl nitrate, and (*E*)- and (*Z*)-2-methyl-4-hydroxybut-2-enyl nitrates (‘isoprene nitrates’) in 66–80% overall yields. Using NOESY experiments the elucidation of the carbon–carbon double bond configuration within the purified isoprene nitrates has been established. Further exemplifying our ‘halide for nitrate’ substitution chemistry we outline the straightforward transformation of (1*R*,2*S*)-(−)-myrtenol bromide into the previously unknown monoterpene nitrate (1*R*,2*S*)-(−)-myrtenol nitrate.

## Introduction

Understanding the chemistry of the biosphere and its interaction with the atmosphere is fundamental to Earth System science. Such is the importance of this topic a whole issue of *Chemical Reviews* was dedicated to the ‘Role of Chemistry in the Earth’s Climate’ [1].

Plants emit into the biosphere a substantial amount (0.5–2%) of their assimilated carbon as small organic molecules. In this context isoprene is one of the most important compounds emitted [2], however, many other different types of biogenic volatile organic compounds (BVOCs) are also generated and released. These include monoterpenes, e.g., 1,8-cineole, borneol, β-phellandrene, 2-carene, camphene, sabinene and citral; sesquiterpenes, e.g., α-copaene, β-cubebene, α-cedrene, β-selinene, α-farnesene, β-gurjunene, β-muurolene and *allo*-aromadendrene, as well as simple alkanes, alkenes, alcohols, esters, aldehydes, ketones and carboxylic acids [3].

The quantities of BVOCs released far exceed non-methane hydrocarbon emissions derived from anthropogenic activity (~90 Tg C yr^−1^ where 1 Tg = 1 × 10^6^ tons) [4]. Indeed, although early estimates of isoprene emissions were as high as 1200 Tg C yr^−1^ [5] these have, in recent years, been revised down to 600 Tg C yr^−1^ [6]. Be this as it may, a comprehensive understanding of the atmospheric chemistry and kinetics associated with isoprene and its derivatives is largely based on chemical theory and modeling with very little verified measurements. Therefore, the impact of isoprene on air quality and climate change remains highly uncertain.

Isoprene or 2-methylbuta-1,3-diene is a volatile C_5_-organic compound generated by plants to help protect them against ’attack’ from bacteria, fungi and parasites. Isoprene also helps to protect against abiotic ’stress’ induced by excessive fluctuations in temperature, by drought, exposure to radiation as well as by contact with herbicides and insecticides [7]. Isoprene reacts readily with O_3_, HO^•^ and ^•^NO_3_ with the resulting intermediates subsequently reacting with NO_X_, i.e., nitric oxide and nitrogen dioxide in a process that generates mixtures of isoprene nitrates (IPNs), O_3_ and secondary organic aerosols (SOA) [8]. Thus, the formation of IPN mixtures plays a key role in O_3_ synthesis and it is this aspect of their chemistry that determines how much NO_X_ is lost or recycled [9].

**Scheme 1:**
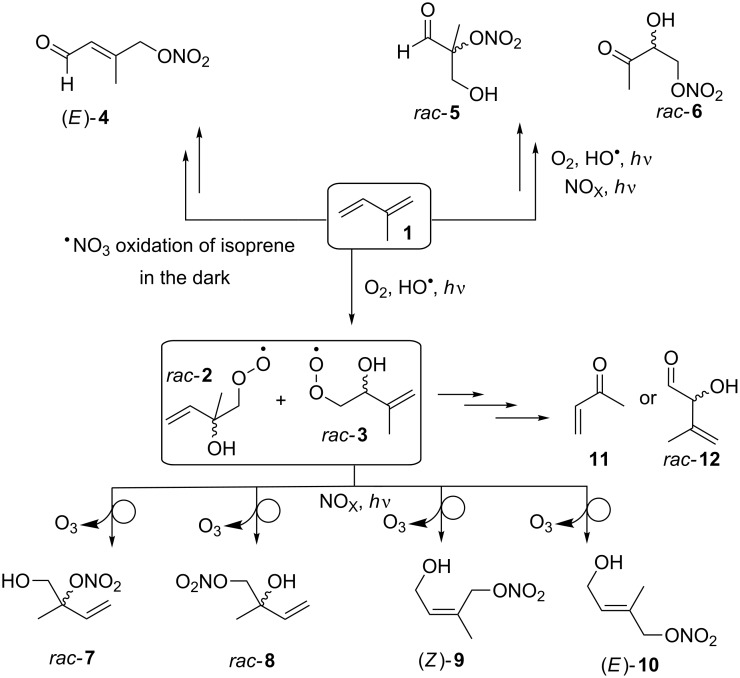
Simplified overview outlining how a small number of different IPNs are synthesised and are able to undergo transformations within the atmosphere.

Taking all of this into account it is clear that isoprene and isoprene nitrates are important. However, what is still not fully understood is the role of individual IPNs (Scheme 1) on atmospheric chemistry [1–3]. Evidently, to comprehensively investigate the role of individual IPNs within climate chemistry it is important that any synthetic protocol employed to generate IPNs affords either individual C=C stereoisomers or generates readily separable stereoisomers of the IPNs. Thus although the lack of synthetic standards has hindered a comprehensive understanding of IPN climate chemistry, there has been progress in a number of laboratory, field and theoretical studies that have focused on probing their formation, kinetics, yields and decomposition. By way of example, Teng et al. investigated the branching ratio (α) when C_2_–C_8_ alkenes (including isoprene) react with HO^•^ in the presence of oxygen. In this process the generated β-hydroxyperoxy radicals subsequently react with NO affording β-hydroxy nitrates. The results from the Teng laboratory established that increasing the substitution pattern on the alkene affords a higher α or branching ratio. A further interesting observation was identified when deuterated alkenes were employed, in these examples α increased by a factor of ~1.5 [10].

Within the atmosphere a wide range of struturally diverse IPNs have been identified. However, the formation of IPN mixtures has led to uncertainty in quantifying the true effect of isoprene on the NO_x_ cycle and subsequent O_3_ enhancement. Shepson et al. sought to deconvolute this process replicating the atmospheric synthesis of IPN using a photochemical reaction chamber to determine IPN yield from isoprene photooxidation and high NO concentration. They compared their results with field observations, collected during the Southern oxidant and Aerosol Study (SOAS) campaign conducted in 2013, and model simulations. These studies identified NO as the limiting factor in IPN production [11].

Schwantes et al. reported a comprehensive study that focused on the oxidation of isoprene with a nitrate radical. Using a variety of detection methods, e.g., CIMS and GC-FID they identified the nitrate radical preferentially reacted at the C1 position of isoprene. The resulting intermediate subsequently reacted with oxygen affording a suite of nitroxyalkylperoxy radicals. Worthy of note and a fundamental reason for initiating the UEA study was the fact that Schwantes et al. make reference to the fact that “synthetic standards are not available, the CIMS sensitivities for most of the isoprene nitrates formed in this work are not known“ [12].

Organic nitrates are important in locations where biogenic hydrocarbon emissions mix with anthropogenic NOx sources. It is generally accepted that transport models should include representation of organic aerosols derived from the reaction of monoterpenes with nitrate radicals. With this in mind Pye et al. recently developed a system to study the formation and subsequent aerosol-phase partitioning of organic nitrates derived from both isoprene and monoterpenes. Their studies indicated the concentrations of organic aerosol and gas-phase organic nitrates increased when particulate organic nitrates underwent rapid pseudohydrolysis; a process that generates corrosive nitric acid and non-volatile SOA [13].

Similar to the isoprene studies by Pye et al. the role of organic nitrates derived from terpenes is starting to gain traction. Rindelaub et al. undertook a photochemical reaction chamber study that focused on the hydroxyl radical oxidation of α-pinene under high NO_x_ conditions. α-Pinene is an important contributor to SOA with annual emissions estimated to be 66 Tg C yr^−1^. In their study using nitric oxide (NO) the yield of α-pinene derived organic nitrate was determined to be 26 ± 7%, interestingly the concentration of organic nitrates was found to be highly dependent on the relative humidity and seed aerosol acidity. Worthy of note this report also highlighted that “unfortunately, standards are unavailable for the expected organic nitrate products (derived from *α*-pinene)“ this, again, reinforces the need for chemical synthesis studies on the formation of climate relevant organic nitrates [14].

Even though the atmospheric synthesis of structure diverse C_5_-IPNs, i.e., **4–10** is thought to be efficient, i.e., up to 15% yield [15] what is not currently fully understood is the mechanism or kinetics of IPN formation, the percentages of individual IPNs generated or how they are synthesised and the products of their decomposition. Answering these important questions requires the combined expertise of synthetic and atmospheric chemists using the former to generate authentic samples of IPNs which can be ’handed over’ to the atmospheric chemists for subsequent testing.

A comprehensive and systematic approach to bespoke IPN synthesis has yet to be undertaken. Here we outline preliminary results towards the development of a series of efficient synthetic routes to IPNs that should allow atmospheric chemists to undertake physicochemical analysis of important climate related IPNs.

In the atmosphere isoprene (**1**) has a half-life of ~1−2 hours [16]. It reacts readily with HO**^•^** and O_2_ generating alkoxy radical (RO**^•^**) intermediates (not shown) as well as hydroperoxy radicals (ROO**^•^**) such as *rac-***2** and *rac-***3** (Scheme 1). The formation and reaction of these reactive intermediates with NO generates O_3_ and mixtures of IPNs, i.e., *rac*-**7**–(*E*)-**10** (Scheme 1). Contributing to the complexity of the climate chemistry associated with **1** is its oxidation to ketones, e.g., **11** and *rac-***6** and aldehydes, e.g., *rac-***5**, *rac-***12** and (*E*)-**4**.

**Scheme 2:**
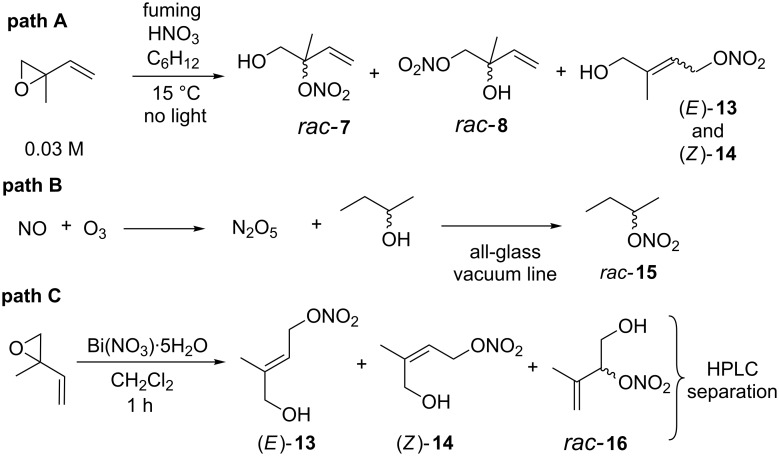
Protocols for the synthesis of *O*-nitrated alcohols using (±)-isoprene epoxide and 2° alcohols as starting materials.

A comprehensive survey of the literature revealed three general synthesis routes to IPNs. In summary, Shepson et al. [17] reacted isoprene epoxide with concentrated nitric acid (Scheme 2, path A); Kames et al. outlined the *O*-nitration of simple alcohols using dinitrogen pentoxide [18] (Scheme 2, path B); Cohen et al. reported the application of bismuth(III) nitrate for isoprene epoxide ring-opening/trapping with nitrate [19] (Scheme 2, path C).

The 2010 report by Shepson et al. (path A) exploited chemistry originally described by Nichols et al. who, employing nitric acid as a convenient and cheap Brønsted acid, transformed a range of epoxides [20] into the corresponding nitrato alcohols. Shepson substituted ethylene oxide for commercially available isoprene epoxide and generated eight stereo- and structurally isomeric IPNs. Within this mixture 3° nitrate *rac*-**7**, 1° nitrate *rac*-**8**, (*E*)-**13** and (*Z*)-**14** (path A, Scheme 2) were generated and separated in the following ratios 31.5:2.43:2.14:1. From a purely practical point of view the addition of ’fuming’ nitric acid to isoprene epoxide is relatively straightforward. However, several drawbacks to using this protocol are immediately evident. Not least the fact that dispensing strongly oxidizing ’fuming’ (i.e., >90%) nitric acid onto reactive isoprene epoxide affords the distinct possibility that a dangerous exothermic ‘runaway’ reaction could take place (cautionary note: experimental section labelled this reaction “highly exothermic”) or indeed an explosion if heated (cautionary note: experimental reports 2-nitratoethanol underwent a “violent explosion, possibly detonation”). Furthermore, although the reaction afforded gram quantities of the IPN mixture the separation and purification of this into *rac-***7**, *rac-***8**, (*E*)-**13** and (*Z*)-**14** was restricted to 100 μL aliquots. Furthermore this did not afford the individual products in high purity. Consequently an additional analytical HPLC purification was required for each ’semi-pure’ fraction using a substantially smaller 20 μL column. In summary, this route was time consuming, labour intensive and expensive affording only small quantities of IPNs.

In 1993 Kames et al. [18] described the reaction of dinitrogen pentoxide with seventeen low molecular weight alcohols. They demonstrated that dinitrogen pentoxide reacted readily with, for example, 1-propanol, 1-hexanol, *rac*-2-pentanol, 1-octanol and that *rac*-2-butanol afforded *rac-*2-butyl nitrate (*rac-***15**, path B, Scheme 2) although no yields were provided. Unfortunately this protocol requires the pre-synthesis of dinitrogen pentoxide from nitric oxide and 5% ozone in oxygen. Evidently the handling and reaction of different gases as well as the synthesis of dinitrogen pentoxide necessitates access to or the construction of a specialist gas manifold linked up to a pirani-gauge, capacitance-pressure transducers and an ozone generator. Additionally once generated the dinitrogen pentoxide requires purification via sublimation and low temperature recrystallization under a continuous stream of ozone and oxygen. Not only is this a time-consuming process it also requires considerable experimental expertise. Furthermore, although using dinitrogen pentoxide is suited to the small-scale synthesis of volatile *O*-nitrate esters, such as *rac-***15**, its application to the synthesis of high-boiling diols is more difficult due to their low-vapour pressures.

Recently Cohen et al. synthesised mixtures of IPNs via the reaction of (±)-isoprene epoxide with pre-ground bismuth(III) nitrate [19]. Using a preliminary ’flash’ purification (silica gel) afforded an improved, but still impure, mixture of (*E*)-**13**, (*Z*)-**14** and 2° *O*-nitrate ester *rac-***16**. Subsequent purification via analytical HPLC afforded small quantities of pure (*E*)-**13**, (*Z*)-**14** and *rac-***16** in an overall yield of approximately 10% and in 7.3:2.7:1 ratio’s respectively (path C, Scheme 2).

The goal of our research program was the development of a ‘suite of protocols’ that afford specific IPNs using straightforward, reliable chemistry. Worthy of note, we also considered the development of efficient synthesis routes to small organic nitrates to have broader benefits to the general synthesis community. By way of example, currently there are very few protocols that afford multi-functional allylic nitrates. This, it would seem, has hindered their exploitation in the development of new synthetic methodology. Furthermore and in a slightly different context there is significant interest in the pharmaceutical sector in generating structure and function diverse *O*-nitrate esters for use as in vivo NO-donors. In this context particular emphasis has been placed on developing *O*-nitrate esters as biologically active agents that act on acetylcholinesterase (AChE), amyloid-βx-42 (Aβ42) aggregation, cyclooxygenase-II (COX2), serotonin reuptake and specific GAG inhibitors [21–23].

## Results and Discussion

**Scheme 3:**
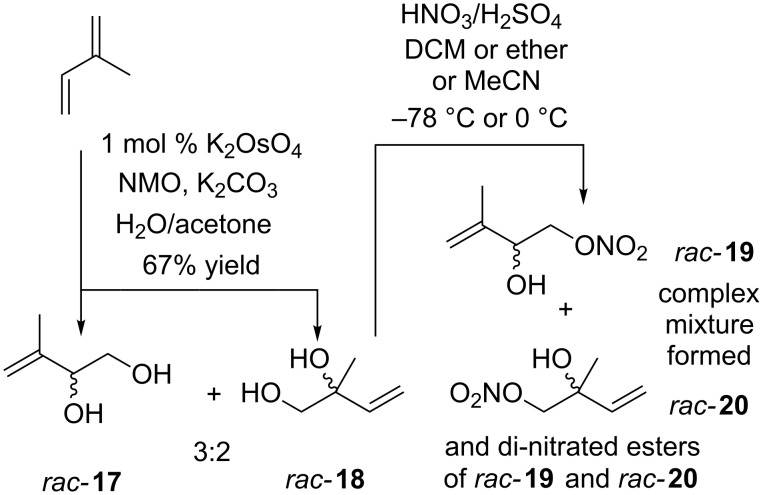
Attempted synthesis of *O*-nitrate ester *rac*-**19** and *rac*-**20** synthesis.

Initiating our research we wanted to generate *O*-nitrate esters based on *rac-***8**, (*Z*)-**9**, (*E*)-**10** (Scheme 1), (*E*)-**13**, (*Z*)-**14**, and *rac*-**16** (Scheme 2). Exploring the potential of isoprene as a starting material we considered it to have several advantages: it is cheap, commercially available, easily handled and has the prerequisite C_5_-skeleton that ensures it is a highly desirable and amenable starting material for its chemical transformation into IPNs. Subjecting isoprene (10 mmol) to a racemic Sharpless dihydroxylation [24] afforded the inseparable (flash chromatography) (±)*-*3-methylbut-3-ene-1,2-diol (*rac-***17**) and (±)*-*2-methylbut-3-ene-1,2-diol (*rac-***18**) in a 3:2 ratio and unoptimized 67% yield (Scheme 3). Investigating *O*-nitrate ester formation the *rac-***17**/*rac-***18** mixture was dissolved in dichloromethane, acetonitrile, or ether at 0 °C or −78 °C. To each was added nitric acid (16 M) and concentrated sulfuric acid (18 M), a biphasic organic/inorganic reaction mixture formed [25]. Unfortunately after work-up all of these reactions afforded complex mixtures. Thus although the ^1^H NMR and mass spectrometry indicated the desired mono-*O*-nitrate *rac-***19** and *rac-***20** had formed (Scheme 3) and it seemed that the corresponding di-*O*-nitrate esters (not shown) had also formed; all attempts at flash chromatographic separation and purification met with failure; a fact that hindered our ability to confidently analyse and identify individual components. In summary, the seemingly straightforward combination of nitric and sulfuric acid was unsuited to the efficient synthesis of IPNs.

**Scheme 4:**
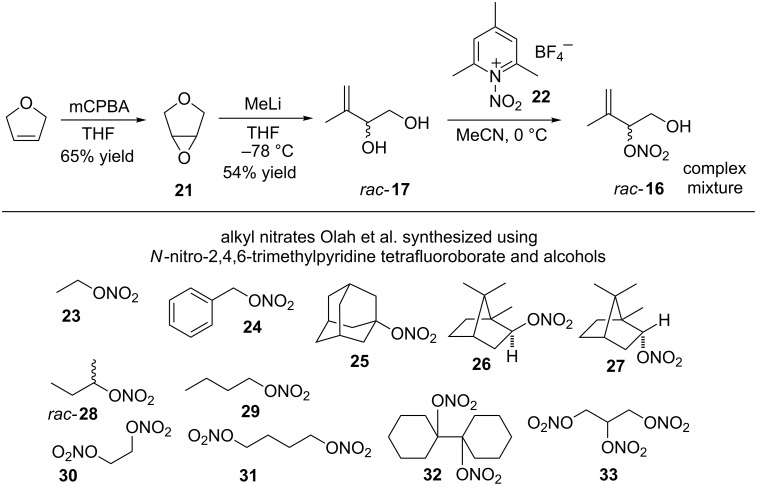
Olah et al. *O*-nitrated alcohol syntheses of **23**–**33** using *N*-nitro-2,4-6-trimethylpyridinium tetrafluoroborate (**22**).

Although generating a mixture of *rac*-**17** and *rac*-**18** was broadly acceptable due to their ease of synthesis, our inability to effectively separate the diols or execute a regioselective 1° or 2° mono-*O*-nitration was not. Eliminating the former problem Hodgson et al. [26] reported *rac*-**17** could be generated from cheap, commercially available 2,5-dihydrofuran which, after epoxidation with *meta*-chloroperbenzoic acid (mCPBA), afforded epoxide **21** in a 65% yield. Subsequent reaction of **21** with methyllithium (2.5 equiv, −78 °C, THF) in an alkylative double ring-opening process afforded, exclusively, (±)-3-methylbut-3-ene-1,2-diol (*rac*-**17**) in a 54% yield (Scheme 4). The next step required the mild regioselective O-nitration of *rac-***17**. Olah et al. reported that nitronium tetrafluoroborate reacts with 2,4,6-trimethylpyridine in acetonitrile at −10 °C affording *N*-nitro-2,4,6-trimethylpyridinium tetrafluoroborate (**22**) [27]. Disappointingly, subjecting *rac-***17** to *O*-nitration with in situ generated **22** resulted, as judged by ^1^H NMR, in a very poor yield (<10%) of a mixture of *O*-nitrate esters that, potentially, also included the desired *rac*-**16**. Indeed, although we explored alternative reaction times, temperatures, solvents and stoichiometry’s of **22** our attempts at generating *rac-***16** were disappointing. Furthermore, employing short or longer reaction times at −78 °C afforded low yields of complex mixtures that proved, essentially, inseparable by flash chromatography.

**Scheme 5:**
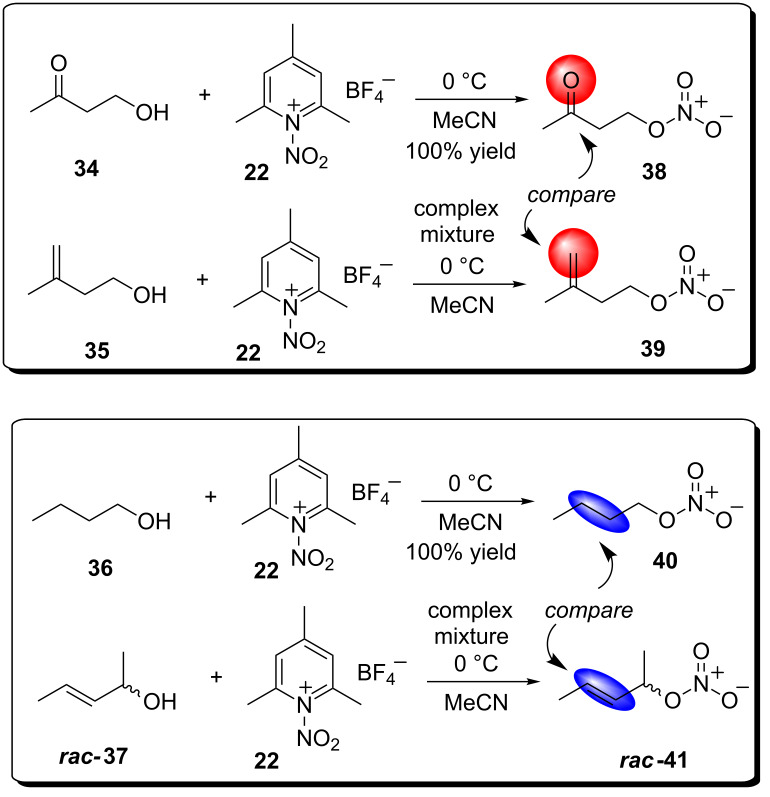
*O*-nitration study using **22** and the alcohols **34**–**37**.

Upon closer inspection of the Olah report we were intrigued by the fact that of the eleven alcohols employed none of the corresponding *O*-nitrate esters, i.e., **23–33** (Scheme 4) contained a non-conjugated ‘isolated’ C=C bond, typical of an allylic alcohol, i.e., *rac*-**17** (Scheme 4). Intrigued by the possibility it was the C=C bond of *rac*-**17** that was contributing, in a negative sense, to a poor reaction outcome a ‘compare and contrast study’ using paired-up alcohols, i.e., **34** (4-hydroxy-2-butanone)/**35** (3-methyl-3-buten-1-ol) and **36** (butan-1-ol)/**37** (*rac*-3-penten-2-ol) were *O*-nitrated using in situ generated **22** (Scheme 5).

Treating **34** and **35**, independently, to the Olah *O*-nitration conditions (MeCN, 0 °C and 1.5 equiv of in situ generated **22**) the desired mono-*O*-nitrated adduct **38** was afforded in a quantitative yield in only two hours. On the contrary C=C bond containing 3-methylbut-3-ene-1-ol (**35**) reacted with **22** (1.5 equiv) affording (^1^H NMR) a complex mixture that contained a small amount of **39** (Scheme 5). Similar to **34**, butanol (**36**) afforded a quantitative yield of *n*-butyl nitrate (**40**) in two hours. Carbon–carbon double bond containing *rac-(E*)-pent-3-en-2-ol (**37**) generated a complex mixture (determined via ^1^H NMR) of, again, largely unidentifiable compounds. Albeit our compare and contrast study was limited to only a handful of simple substrates it did generate convincing evidence that synthesising C=C containing IPNs using **22** was problematic. A potential reason for the inability of **22** to cleanly generate **39** or **41** was associated with the propensity of nitronium salts to mediate alkene polymerization [28]. An alternative further plausible explanation for failure to isolate the C=C derived O-nitrates focuses on a report by Scheinbaum and Dines who established that alkenes in the presence of acetonitrile and **22** undergo a Ritter reaction affording vicinal nitro acetamido species [29].

**Scheme 6:**
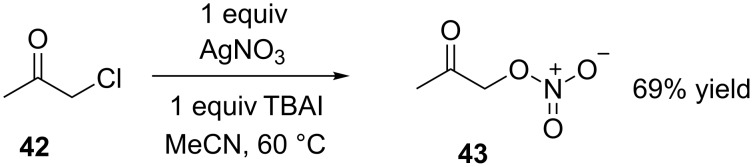
Silver nitrate mediated synthesis of 2-oxopropyl nitrate **43**.

Silver nitrate reacts with alkyl, benzyl or acyl halides affording the corresponding alkyl [30], benzyl [31–32] or acyl nitrates [33]. By way of example and relevant to the work reported here Ogawa et al. [34] transformed bromoacetone to 2-oxopropyl nitrate (component of climate mediated IPN decomposition [35]) using silver nitrate in acetonitrile. Initiating the synthesis of ‘test substrate’ **43** we reacted silver nitrate in acetonitrile with the cheap, commercial and readily available chloroacetone in place of the less accessible and considerably more expensive bromoacetone. After 16 hours at 40 °C the <15% yield of **43** was disappointing; it seemed the enhanced reactivity associated with bromoacetone was a requirement for an efficient ‘bromide to nitrate’ substitution. Changing tactics we opted to generate the more reactive iodoacetone in situ from chloroacetone (**42**) and a stoichiometric quantity of tetra-*n*-butylammonium iodide (TBAI). Gratifyingly the iodoacetone reacted readily with silver nitrate at 60 °C affording **43** (Scheme 6) as a yellow oil in an unoptimized 69% yield. Unfortunately the unstable and reactive properties of **43** meant purification was not straightforward [36]. However installation of the nitrate group was confirmed via comparison of our data with that in the literature [28]. Thus in the ^1^H NMR the downfield shift of the -CH_2_Cl group from 4.15 ppm to 4.94 ppm was associated with the installation of NO_3_ affording the -CH_2_ONO_2_ group. With this positive result in hand application of the halide to nitrate transformation within the context of IPN synthesis was initiated. Interestingly, although silver nitrate has been widely employed for the synthesis of structure and function diverse benzyl nitrates; the analogous reaction affording allylic nitrates using allylic halide starting materials has, surprisingly, been reported only once [37].

**Scheme 7:**
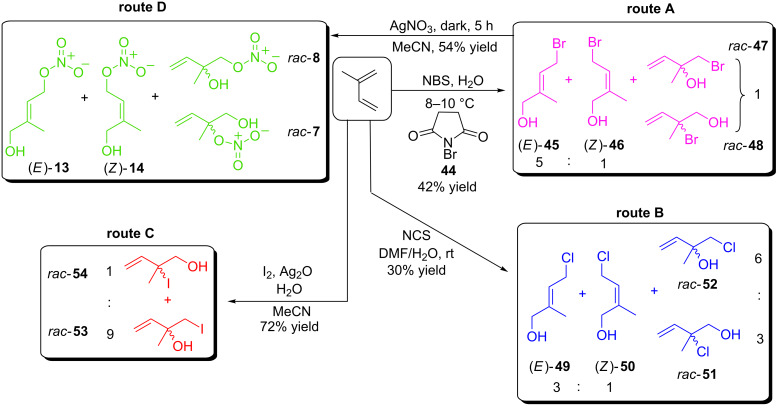
Application of isoprene for the synthesis of precursors to IPNs and synthesis via ‘halide for nitrate exchange’ of *rac*-**7**, *rac*-**8**, (*E*)-**13** and (*Z*)-**14**.

The lack of prior art associated with the synthesis of (*E*)-2-methyl-4-bromobut-2-en-1-ol validated the importance of this seemingly valuable starting material and useful building block. Indeed it is not commercially available and has been reported in the patent literature only once. Following the procedure of Gurumurthy et al. [38] isoprene was reacted with *N*-bromosuccinimide (NBS, **44**) in water at 8–10 °C for 2 h. The reaction afforded a mixture of two allylic bromides and two allylic alcohols in a combined 42% yield, these were tentatively assigned as (*E*)-**45**, (*Z*)-**46**, *rac-***47** and *rac-***48** (route A, Scheme 7). All attempts at separating this mixture using flash column chromatography were unsuccessful. Repeating this reaction but with *N*-chlorosuccinimide (NCS, route B, [39]) or alternatively using iodine and silver(I) oxide (route C, [40]) we considered the possibility that the resulting allylic chlorides or iodides may be more amenable to separation. Utilizing a procedure reported by Koo et al. [39] isoprene and NCS were stirred in aqueous DMF at ambient temperature for 4 hours. ^1^H NMR analysis indicated that (*E*)-**49**, (*Z*)-**50**, *rac-***51** and *rac-***52** had formed. Disappointingly our attempts at isolating and purifying the individual components were only partially successful. Thus although *rac-***51** and *rac-***52** could be separated, (*E*)-**49** and (*Z*)-**50** could not.

Disappointed with this outcome we opted to react isoprene with a combination of iodine and silver(I) oxide in aqueous acetonitrile. Our intention was to generate a mixture of, hopefully, separable allylic alcohols and allylic iodides. Interestingly this alternative protocol afforded only two of the possible four products. Thus terminal alkene derived 1-iodo-2-methylbut-3-en-2-ol (*rac*-**53**) and 2-iodo-2-methylbut-3-en-1-ol (*rac*-**54**) were afforded in a 9:1 ratio, respectively, and a pleasing 72% yield (route C, Scheme 7). Analysis via ^1^H NMR afforded no evidence for the formation of (*E*)- or (*Z*)-2-methyl-4-iodo-2-but-2-en-1-ol. Needless to say the formation of *rac-***53** and *rac-***54** was of little benefit as their separation proved impractical via flash column chromatography.

As noted (Scheme 2) the efficient separation of (*E*)-**13** and (*Z*)-**14** was not possible without recourse to analytical HPLC [9]. Nevertheless we considered it important to validate our proposed ‘halide for nitrate’ substitution by undertaking a ‘test’ reaction using silver nitrate and the allylic bromide/allylic alcohol mixture (*E*)-**45**–*rac-***48**. Dissolving this in acetonitrile the reactants were protected from light by wrapping the flask in aluminium foil and one equivalent of silver nitrate was added. The reaction was stirred for 5 hours at ambient temperature after which the reaction was complete, a simple filtration removed the silver bromide that had generated. Subsequent solvent removal and analysis via ^1^H NMR indicated the IPNs had formed. Subjecting the mixture to flash column chromatography and, similar to previous reports, it was not possible to separate (*E*)-**13**, (*Z*)-**14**, *rac-***7** or *rac-***8** and it was therefore not possible to determine, unambiguously, if all of the above had been formed. Nevertheless physicochemical analysis of the ‘purified’ mixture (54% yield) indicated the *O*-nitrate esters were present with distinctive FTIR absorption peaks located at 1635 and 1278 cm^−1^. Furthermore changes in the ^1^H NMR chemical shifts compared to the starting materials as well as GC–MS analysis (negative ion mode) corroborated the *O*-nitrate ester groups were present.

**Scheme 8:**
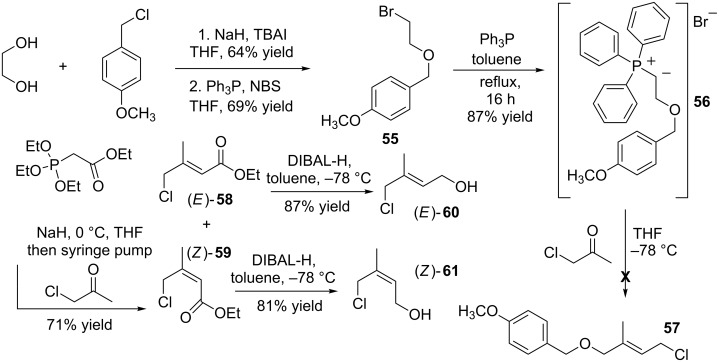
Synthesis of (*E*)-3-methyl-4-chlorobut-2-en-1-ol ((*E*)-**60**) and (*Z*)-3-methyl-4-chlorobut-2-en-1-ol ((*Z*)-**61**).

Although the use of silver nitrate had been validated in our ‘test’ reaction, the use of isoprene as a starting material was clearly not as convenient as first envisaged. Its application was restricted by its propensity to generate difficult to separate structure and stereoisomeric mixtures of allylic bromides and alcohols, e.g., (*E*)-**45**–*rac-***48**. We contemplated using Wittig or Horner–Wadsworth–Emmons (HWE) chemistry to construct (*E*)-alkyl 3-methyl-4-chlorobut-2-enoates which [41–43] possessing a chemically differentiated C=C bond (appended at one end with a chloromethylene and the opposing end an ester) should allow the chemoselective reduction of the ester to the corresponding 1° alcohol. This we predicted would allow, depending on the starting material employed, the synthesis of either (*E*)-3-methyl-4-chlorobut-2-en-1-ol ((*E*)-**60**) or (*Z*)-3-methyl-4-chlorobut-2-en-1-ol ((*Z*)-**61**, Scheme 8). Reacting triphenylphosphine with 1-((2-bromoethoxy)methyl)-4-methoxybenzene (**55**) generated non-stabilized phosphonium ylide (2-(4-methoxybenzyloxy)ethyl)triphenylphosphonium bromide (**56**), we envisaged its subsequent deprotonation and addition to chloroacetone would afford (*E*)-1-((2-methyl-4-chlorobut-2-enyloxy)methyl)-4-methoxybenzene (**57**). Inclusion of the PMB-ether was beneficial due to the ease with which it can be cleaved using readily available reagents, e.g., DDQ or CAN and mild conditions [44]. The synthesis of the previously unknown **56** [45] was high yielding, i.e., 87% yield (Scheme 8). However despite numerous attempts employing different bases [46], e.g., NaH, LHMDS, *t*-BuOK, as well as reaction temperatures, e.g., 0 °C and −78 °C and solvents, e.g., THF and ether all our efforts at generating **57** failed, affording instead highly coloured, complex mixtures (^1^H NMR) that were difficult to purify. Whilst researching alternative organophosphorous mediated C=C bond forming reactions that incorporated chloroacetone the synthesis of 3-methyl-4-chlorocrotonic ethyl ester via a Horner–Wadsworth–Emmons reaction caught our attention [47]. Changing tack and in a slightly modified procedure to that originally reported by Fujiwara et al. triethyl phosphonoacetate was deprotonated (NaH) and the resulting stabilised ylide (not shown) reacted by slow addition of chloroacetone in THF. It was important in establishing high yields of (*E*)-**58** and (*Z*)-**59** to use a syringe pump. This helped to minimise the number and amounts of side-products formed via, presumably, the enolate of chloroacetone which likely undergoes rapid secondary reactions. Subsequent work-up and purification afforded stereoisomers (*E*)-**58** and (*Z*)-**59** in a 2:1 ratio, respectively, and combined, unoptimized, 71% yield.

Separation of the stereoisomers via flash column chromatography was straightforward. Pure (*E*)-**58** and (*Z*)-**59** were afforded with physicochemical properties essentially identical to those reported [48]. Taking (*E*)-**58** and (*Z*)-**59** forward their reduction to (*E*)-**60** and (*Z*)-**61** using DIBAL-H (−78 °C) was uncomplicated. The corresponding (*E*)- and (*Z*)-allylic alcohols were afforded in 87% and 81% yields, respectively. The physicochemical properties of (*E*)-**60** were essentially identical to those reported [49]. The potential for (*E*)-3-methyl-4-chlorobut-2-en-1-ol ((*E*)-**60**) to react with silver nitrate generating (*E*)-**10** (Scheme 1) was investigated. Dissolving it in acetonitrile, the flask was wrapped in aluminium foil and one equivalent of silver nitrate added. After stirring for 16 hours at ambient temperature a sample was removed for ^1^H NMR analysis. This indicated approximately 15% of (*E*)-**60** had been consumed. Enhancing the sluggish reactivity of (*E*)-**60** a catalytic amount of sodium iodide (10 mol %) was added (presumably generating in situ the corresponding allylic iodide). After stirring for a further 16 hours, the majority of (*E*)-**60** had reacted as judged by TLC and ^1^H NMR analysis. Removing the sodium chloride via a simple filtration was more advantageous than the previously employed organic soluble and more expensive TBAI (Scheme 6). Straightforward flash-column purification afforded (*E*)-**10** in an unoptimized 60% yield and with physicochemical properties similar to those reported [9].

**Scheme 9:**
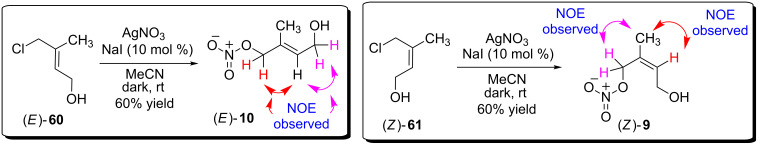
Using NOESY interactions to establish the conformations of the C=C bonds within (*E*)-**10** and (*Z*)-**9**.

Confident our silver nitrate mediated halide substitution protocol was robust, attention switched to incorporating (*Z*)-3-methyl-4-chlorobut-2-en-1-ol ((*Z*)-**61**). Employing a sodium iodide enhanced reaction (10 mol %) using 16 mmol of (*E*)-**61** afforded 1.4 g of (*Z*)-2-methyl-4-hydroxybut-2-enyl nitrate ((*Z*)*-***9**) in an unoptimized 60% yield. Comparing the ^1^H NMR data associated with (*E*)-**10** and (*Z*)-**9** revealed they were, as expected, broadly similar (see Supporting Information File 1). However, subtle chemical shift differences were evident, most notably with those associated with the CH_3_ attached to the C=C bond, i.e., 1.75 ppm ((*E*)-**10**) and 1.84 ppm ((*E*)-**9**); similarly the CH_2_ONO_2_ groups were located at 4.84 ppm for (*E*)-**10** and 4.97 ppm for (*Z*)-**9**. Substantiating our tentative carbon–carbon double bond stereochemical assignment for (*Z*)-**9** and (*E*)-**10** was important. Homonuclear two-dimensional NOE spectroscopy (NOESY) was used to explore the C=C double bond configuration. (*E*)-**10** displayed two NOE interactions between the alkene proton and the methylene groups of the adjacent -CH_2_OH and -CH_2_ONO_2_ (see ‘double-headed arrows’, Scheme 9). In (*Z*)-**9** the alkene proton had an NOE interaction with the adjacent CH_3_ (see red arrow, Scheme 9) and the methylene of the -CH_2_ONO_2_ group likewise had an observable interaction with the CH_3_ (see purple arrow). However, unlike (*E*)-**10** no interaction was observed between the alkene proton and the methylene of the -CH_2_OH group. Importantly, the lack of an interaction between the (*E*)-C=C bound hydrogen on **10** and the ‘(*E*)-CH_3_‘ group afforded good evidence that **10** was indeed a (*E*)-configured C=C bond.

Furthermore comparison of our ^1^H NMR spectra with the one reported by Lee et al. [19] for structural isomer (*E*)-4-hydroxy-3-methylbut-2-enyl nitrate ((*E*-)**13**, Scheme 2) displayed a subtle chemical shift difference centred on the methyl group attached to the C=C bond. By way of example, (*E*)-**13** displayed the methyl at 1.75(7) ppm whilst we observed the methyl group in (*E*)-**10** as a sharp singlet at 1.69 ppm, perhaps more importantly the alkene proton of silver nitrate generated (*E*)-**10** was identified as a triplet (*J* = 5.8 Hz) at 5.73 ppm compared with 5.64 ppm (*J* = 7.2 Hz) for (*E*)-**13**. We attempted to further substantiate our assignment by undertaking a structure search on SciFinder for (*E*)-**10** comparing the ^1^H NMR data available with ours. However, although 5 papers report (*E*)-**10** none, unfortunately, had any ^1^H NMR data.

A SciFinder search for (*Z*)-**9** afforded 3 references, similar to (*E*)-**10**, none reported any ^1^H NMR data that could be used as reference spectra. In addition to (*E*)-**13** Lee et al. described [19] the synthesis and ^1^H NMR of (*Z*)-**14** the structural isomer of (*Z*)-**9**. Comparing their physicochemical data sets similar but subtle differences were evident. Thus the alkene proton in (*Z*)-**14** was observed as a triplet (*J* = 7.6 Hz) at 5.45(7) ppm whilst in our synthesised (*Z*)-**9** the similarly positioned proton was also observed as a triplet but with a smaller coupling constant, e.g., *J* = 6.4 Hz located at 5.82 ppm.

**Scheme 10:**
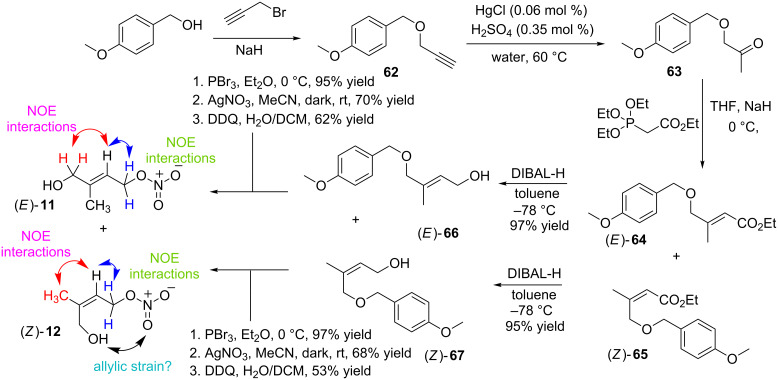
Synthesis of isoprene nitrates (*E*)-**11** and (*Z*)-**12** from ketone **63**.

Our preliminary ‘halide for nitrate’ results using silver nitrate and allylic chlorides (*E*)-**60** and (*Z*)-**61** were positive and firmly established this route as a straightforward method of generating stereochemically pure IPNs (*E*)-**10** and (*Z*)-**9**. It was important to complete this preliminary study synthesizing (*E*)-**11** and (*Z*)-**12** (Scheme 10), both of which are structural isomers of (*E*)-**10** and (*Z*)-**9** (Scheme 9). Employing our HWE approach 1-(4-methoxybenzyloxy)propan-2-one (**63**) was easily generated via a two-step protocol (overall 63% yield) that started with the etherification of sodium *para*-methoxybenzyl alcolate with propargyl bromide [50]. The terminal alkyne on **62** was efficiently transformed into a ketone via an oxymercuration reaction using a combination of mercury(I) chloride (0.06 mol %) and sulfuric acid (0.35 mol %) in water following the procedure of Boger et al. [51]. **63** was afforded in an unoptimized 78% yield. Employing the conditions outlined in Scheme 10 **63** reacted with the stabilized ylide generated from the deprotonation of triethyl phosphoacetate with sodium hydride. A separable mixture of (*E*)-**64** and (*Z*)-**65** (1.35:1) was afforded in an overall 61% yield from **62**.

DIBAL-H readily reduced the ethyl ester on (*E*)-**64** and (*Z*)-**65** (−78 °C, toluene) affording 1° alcohols (*E*)-**66** and (*Z*)-**67** in 97% and 95% yields, respectively. Increasing the electrophilic nature of the desired allylic halides (viz. use of allylic chloride and 10 mol % sodium iodide in Scheme 9) we opted to transform 1° alcohols (*E*)-**66** and (*Z*)-**67** into their corresponding allylic bromides (not shown). This was straightforward and efficient using phosphorus tribromide in ether at 0 °C. The desired (*Z*)- and (*E*)-allylic bromides were generated in 95% and 97% yields, respectively. Although the allylic bromides were readily purified (flash column chromatography) their subsequent reaction with silver nitrate had to be undertaken quickly and, ideally, straight away because of their propensity to decomposition. Gratifyingly, reacting (*E*)-1-((2-methyl-4-bromobut-2-enyloxy)methyl)-4-methoxybenzene and (*Z*)-1-((2-methyl-4-bromobut-2-enyloxy)methyl)-4-methoxybenzene with silver nitrate in acetonitrile afforded (*E*)-4-(4-methoxybenzyloxy)-3-methylbut-2-enyl nitrate (70% yield) and (*Z*)-4-(4-methoxybenzyloxy)-3-methylbut-2-enyl nitrate (68% yield) as stable, colourless oils. Mild oxidative cleavage of the PMB groups using DDQ in wet DCM generated the desired 1° allylic alcohol (*E*)-3-methyl-4-hydroxybut-2-enyl nitrate ((*E*)-**11**) and (*Z*)-3-methyl-4-hydroxybut-2-enyl nitrate ((*Z*)-**12**) in 62% and 53% yields, respectively (Scheme 10). Analysing the configuration of the C=C bond in (*E*)-**11** and (*Z*)-**12** via NOESY confirmed, similar to (*E*)-**10** and (*Z*)-**9**, the C=C bonds were, as expected, in the (*E*)- and (*Z*)-configurations for **11** and **12** respectively. Further confirmation of these assignments was sought. Referencing our data with that reported by Lee et al. [19] we were delighted that (*E*)-**11** and (*Z*)-**12** displayed, within experimental error, identical ^1^H NMR spectra. Of note we observed the isomerization of (*Z*)-**12** to (*E*)-**11** to be rapid (1–2 hours), a fact that contrasted quite sharply with the rate of isomerization for (*Z*)-**9** to (*E*)-**10** which was, comparatively, quite slow (~24 hours). Presumably the increased rate of isomerization for (*Z*)-**12** to (*E*)-**11** was associated with relief of the allylic strain between the (*Z*)-configured, polar -CH_2_OH and -CH_2_ONO_2_ groups that reside on the same side of the C=C bond (Scheme 10).

**Scheme 11:**
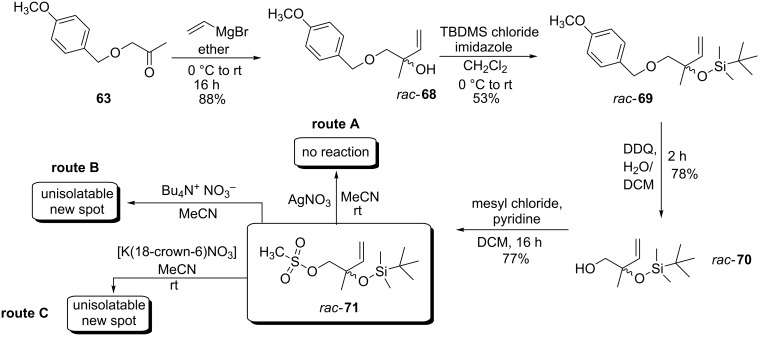
Attempted synthesis of *rac*-**8** from *O*-mesylate *rac-***71**.

The low cost ($1 per gram), ease of use and convenient handling associated with silver nitrate coupled with its straightforward ability to generate (*Z*)-**9–**(*Z*)-**12** convinced us to explore the synthesis of *rac*-**7**, *rac*-**8** (Scheme 1) and *rac*-**16** (Scheme 2). Employing ketone **63** as a readily available ‘core’ starting material its reaction with vinylmagnesium bromide afforded racemic 3° allylic alcohol *rac-***68** in an 88% yield. Subsequent hydroxy group protection using *tert*-butyldimethylsilyl chloride and imidazole afforded orthogonally protected *O*-TBDMS/PMB ether *rac*-**69** in a moderate 53% yield. Needless to say the moderate yield was not problematic as *rac*-**68** and *rac*-**69** were readily separable, allowing *rac*-**68** to be recycled (based on recovered starting material the yield was almost quantitative). Oxidative *O*-PMB deprotection of *rac*-**69** using DDQ in biphasic dichloromethane/water generated 1° alcohol (±)-2-(*tert*-butyldimethylsilyloxy)-2-methylbut-3-en-1-ol (**70**) in a 78% yield (Scheme 11).

**Scheme 12:**
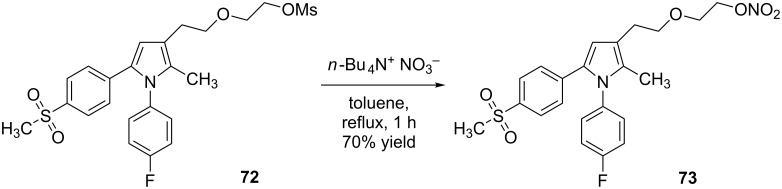
Synthesis of *O*-nitrate **73** from *O*-mesylate **72**.

Attempted conversion of the 1° alcohol on *rac*-**70** into the corresponding 1° alkyl bromide failed to generate the desired product, instead an intractable tar was formed. Changing our approach Anzini et al. [22] demonstrated tetra-*n*-butylammonium nitrate [17] to be a source of nitrate that was capable of efficiently mediating an S_N_2 ‘1° mesylate for nitrate’ substitution, i.e., **72** to **73** (Scheme 12).

Generating the 1° mesylate of *rac*-**70** using methanesulfonyl chloride and pyridine afforded *rac*-**71** in a 77% yield after 16 hours at ambient temperature. Unfortunately subjecting it to a ‘mesylate for nitrate’ substitution akin to our previously successful ‘halide for nitrate’ substitution, cf. (*E*)-**60** to (*E*)-**10** (Scheme 9) using silver nitrate did not work and starting material *rac-***71** was returned in a quantitative yield. Furthermore, we did consider employing the elevated reaction conditions (110 °C) reported by Anzini et al. but were not confident that the *O*-TBDMS protected *O*-nitrate ester *rac*-**8** if formed would be stable [8]. For this reason we explored transforming *rac*-**71** into *O*-TBDMS protected *rac*-**8** at ambient temperature using silver nitrate in acetonitrile. Unfortunately in contrast to previous successful ‘halide for nitrate’ transformations no reaction was observed with mesylate *rac-***71** even after extended reaction times. Repeating the reaction but substituting the silver salt for tetra-*n*-butylammonium nitrate TLC analysis did indicate, after 24 hours, a small percentage (i.e., ~10%) of *rac*-**8** had formed with the remainder comprising *rac*-**71**. However using flash column chromatography to separate *rac*-**71** from the, presumed, 1° nitrate ester was not possible.

Circumventing the lack of reactivity displayed by *rac*-**71** the in situ synthesis of a ‘naked’ nitrate containing species, i.e., [K(18-crown-6)NO_3_] and its application to mesylate displacement was attempted. Premixing potassium nitrate and 18-crown-6 we envisaged would sequester the potassium cation [52] and generate a [K(18-crown-6)NO_3_] complex. Using acetonitrile as solvent one equivalent of [K(18-crown-6)NO_3_] was stirred with *rac*-**71**. Disappointingly, although a new compound was generated it accounted for only a small percentage of the reaction mixture. The majority of *rac*-**71** had not reacted and due to purification issues this route to *O*-nitrate ester synthesis was abandoned.

Disappointed with the lack of reactivity displayed by *rac*-**71** we opted instead to tackle the synthesis of (±)-2-hydroxy-3-methylbut-3-enyl nitrate (*rac*-**19**). Starting from 3-methyl-2-buten-1-ol its conversion, on a multigram scale, to (±)-3-(bromomethyl)-2,2-dimethyloxirane (*rac*-**74**) was straightforward. Heating *rac*-**74** with a mixture of acetic anhydride and *para*-toluenesulfonic acid (PTSA, 1 equiv) afforded via, presumably, protonated *rac*-**75** ring-opened and *O*-acylated (±)-1-bromo-3-methylbut-3-en-2-yl acetate (*rac*-**76**) in a 58% yield. Isolation, solvent removal and purification of *rac-***76** from the reaction byproducts had to be undertaken swiftly because of its relatively rapid decomposition (10–15 minutes). Negating this, a quick flash column was undertaken and the resulting ~75% pure product was taken on ‘as is’. Employing silver nitrate in our standard reaction conditions afforded a novel compound presumed to be (±)-3-methyl-1-(nitrooxy)but-3-en-2-yl acetate (*rac*-**77**) in an unoptimized 70% yield. The final stage of the synthesis was the hydrolysis of the *O*-acetate ester using mild, slightly basic reaction conditions. Thus using potassium carbonate in methanol a new alcohol was afforded in an excellent 94% yield. Seeking structure confirmation a search on SciFinder confirmed *rac-***19** had been previously reported. However inspection of these manuscripts and, more importantly, their electronic supporting information’s established that no ^1^H or ^13^C NMR data associated with *rac-***19** was available.

**Scheme 13:**
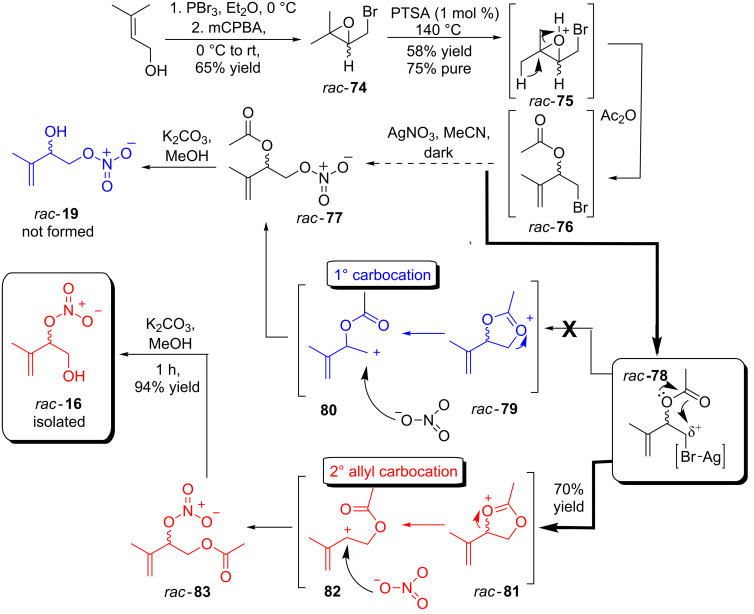
Attempted synthesis of 2° alcohol containing 1° nitrate ester *rac*-**19** and the unexpected synthesis of (±)-1-hydroxy-3-methylbut-3-en-2-yl nitrate, *rac*-**16**.

It was during these studies that we noticed the ^1^H NMR data, reported by Lee et al., for 1° alcohol *rac*-**16** was, within experimental error, identical to the ^1^H NMR observed for the supposed 2° alcohol *rac*-**19** (Scheme 13). It seemed that during our attempted synthesis of *rac*-**16** we had in fact generated *rac*-**19**. Accounting for this we propose that activation of the 1° alkyl bromide with silver nitrate generates an electrophilic species similar to *rac*-**78** which in the presence of the proximal *O*-acetate generates a 5-membered electrophilic acyloxonium species based on *rac*-**79**/*rac-***81**. The formation of such species from simpler non C=C containing starting materials has been reported and established (^1^H NMR) by Gopius et al. [52]. Accounting for the exclusive formation of *O*-acetate *rac*-**83** the trapping of the more stable 2° allyl carbocation intermediate **82** (generated via ring-opening of *rac*-**81**) with nitrate is preferable to the formation of the higher energy/more unstable 1° carbocation **80** (generated via ring-opening of *rac*-**79**, Scheme 13). Subsequent formation of *rac*-**83** allows its hydrolysis with potassium carbonate in methanol to generate the observed *rac*-**16**.

Isoprene and monoterpenes account for a large percentage of the total emissions that are not non-methane VOCs. Furthermore although monoterpene emissions (127 Tg C yr^−1^) are significant lower than isoprene [6] (viz. 600 Tg C yr^−1^) they are nonetheless gaining in significance and are now considered to be important contributors to climate chemistry [53]. By way of example although α-pinene is slightly less reactive to the hydroxyl radical (HO^•^) than isoprene it does, conversely, have a higher reactivity to O_3_ and nitrate radicals (^•^NO_3_) making its atmospheric oxidation significant with respect to regional tropospheric O_3_ and NO_X_ concentrations. The oxidation products derived from monoterpenes have been demonstrated to be important in generating atmospheric based secondary organic aerosols (SOA’s) that have a significant impact on global radiation [54].

**Scheme 14:**
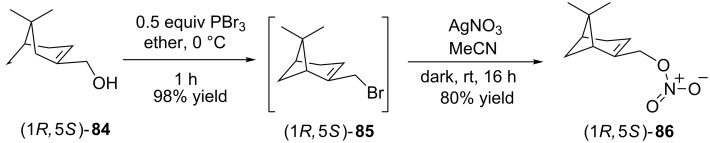
Synthesis of monoterpene derived (1*R*,5*S*)-(−)-myrtenol nitrate **86**.

Exploiting our methodology further its diversity and application to monoterpene derived *O*-nitrate ester synthesis was undertaken using (1*R*,5*S*)-(−)-myrtenol (**84**) which was quickly and efficiently converted into the corresponding optically active 1° alkyl bromide **85** which, due to its instability, was used ‘as is’ [55]. Employing our standard silver nitrate conditions (1*R*,5*S*)-(−)-**85** was efficiently transformed into optically active *O*-nitrate ester (1*R*,5*S*)-(−)-**86** in an unoptimized 80% yield (Scheme 14). The importance of this preliminary result resides in the increased awareness of the role that monoterpene and sesquiterpene nitrates have in climate chemistry. Research, however, on their role is, similar to IPNs, severely limited by the dearth of convenient protocols capable of generating structure and function diverse monoterpene and sesquiterpene nitrates.

Finally, the kinetics and measurement of the IPNs generated within this study and their relationship to aspects of atmospheric chemistry have been reported [56].

## Conclusion

Isoprene, monoterpene and sesquiterpene nitrates are gaining recognition for their significant roles in climate chemistry however an efficient route to their synthesis has yet to be developed. Here we report an efficient silver nitrate mediated synthesis procedure that is broadly applicable to the production of sought-after ‘isoprene nitrates’. The general applicability of this procedure has also been confirmed via its application to the first synthesis of a monoterpene nitrate derived from (1*R*,5*S*)-(−)-myrtenol. In the former examples our protocol proceeds via the application of Horner–Wadsworth–Emmons Chemistry that generates readily functionalized motifs that undergo an all but previously non-existent, allylic ‘halide for nitrate’ substitution reaction. A consequence of the broader importance of organic nitrates we envisage our ‘halide for nitrate’ synthesis transformation will be of considerable interest to, not only atmospheric chemists, but also the wider synthetic and medicinal chemistry communities.

## Supporting Information

File 1 Experimental.
