# Supplementary material for: Efficient syntheses of climate relevant isoprene nitrates and (1R,5S)-(−)-myrtenol nitrate
Source: Beilstein J Org Chem. 2016 May 27;12:1081–95. doi: 10.3762/bjoc.12.103 (PMC4902045; doi:10.3762/bjoc.12.103)
Supplement: File 1 — Experimental. [file Beilstein_J_Org_Chem-12-1081-s001.pdf]

Supporting Information

for

Efficient syntheses of climate relevant isoprene nitrates and (1*R*,5*S*)-(−)-myrtenol nitrate

Sean P. Bew*[1], Glyn D. Hiatt-Gipson[1], Graham P. Mills[2] and Claire E. Reeves[2]

Address: [1]School of Chemistry, University of East Anglia, Norwich Research Park, Norwich, NR4 7TJ, UK and [2]Centre for Ocean and Atmospheric Science, School of Environmental Sciences, University of East Anglia, Norwich Research Park, Norwich, NR4 7TJ, UK

Email: Sean P. Bew - s.bew@uea.ac.uk

*Corresponding author

Experimental

S3–S16 experimental procedures for synthesis of key compounds

S17 ^{1}H NMR and ^{13}C NMR of (*E*)-**58**

S18 ^{1}H NMR and ^{13}C NMR of (*Z*)-**59**

S19 ^{1}H NMR and ^{13}C NMR of (*E*)-**60**

S20 ^{1}H NMR and ^{13}C NMR of (*E*)-**10**

S21 ^{1}H NMR and ^{13}C NMR of (*Z*)-**61**

S22 ^{1}H NMR and ^{13}C NMR of (*Z*)-**9**

S23 ^{1}H NMR and ^{13}C NMR of (*E*)-**64**

S24 ^{1}H NMR and ^{13}C NMR of (*Z*)-**65**

S25 ^{1}H NMR and ^{13}C NMR of (*E*)-**66**

S26 ^{1}H NMR and ^{13}C NMR of (*Z*)-**67**

S27 ^{1}H NMR and ^{13}C NMR of (*E*)-**11**

S28 ^{1}H NMR and ^{13}C NMR of (*Z*)-**12**

S29 ^{1}H NMR and ^{13}C NMR of rac-**74**

S30 ^{1}H NMR and ^{13}C NMR of (*E*)-**83**

S31 ^{1}H NMR and ^{13}C NMR of rac-**16**

S32 ^{1}H NMR and ^{13}C NMR of **86**

S33 references

Synthesis of acetone nitrate (**43)**

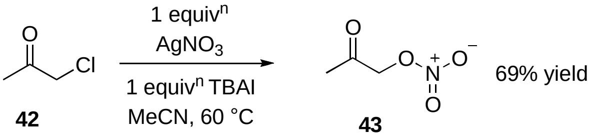

A round-bottomed flask was charged with chloroacetone (**42**, 1.69 g, 18.2 mmol), TBAI (672 mg, 1.8 mmol) and silver nitrate (3.09 g, 18.2 mmol) in acetonitrile (10 mL). The reaction mixture was heated to 60 °C for 16 hours. The precipitate was filtered off and the filtrate transferred to a 25 mL separating funnel. The solution was diluted with water (10 mL) and extracted with diethyl ether (2 × 10 mL). The combined organic extracts were dried over magnesium sulfate, filtered and the solvent removed under reduced pressure affording a pale yellow liquid. Attempts at purifying **43** via flash chromatography failed, excess chloroacetone was removed under reduced pressure affording the title compound 2-oxopropyl nitrate **43** as a colourless liquid (1.5 g, 12.6 mmol, 69%).

^{1}H NMR (500 MHz, $CDCl_3$) δ 4.94 (s, 2H), 2.22 (s, 3H). ^{13}C NMR (126 MHz, $CDCl_3$) δ 197.97, 73.22, 24.97. FT-IR KBr(neat): 2927, 2284, 1722, 1651, 1287 cm^{-1}. m/z GCMS [ES]$^-$ 46 NO_2, 73 $C_3H_5O_2$.

Synthesis of (*Z*)-ethyl 4-chloro-3-methylbut-2-enoate ((*Z*)-**59**) and (*E*)-ethyl 4-chloro-3-methylbut-2-enoate ((*E*)-**58**)

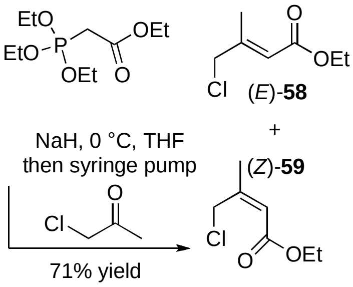

A flame-dried 250 mL round-bottomed flask was charged with THF (50 mL) and cooled to 0 °C in an ice bath. To this sodium hydride (60% in mineral oil, 3.63 g, 91 mmol) was added and left to stir for 5 minutes. Triethyl phosphonoacetate (20 mL, 100 mmol) was added over 1 hour via syringe pump. Following the addition, the solution was allowed to warm to room temperature and left to stir for 1 hour. The solution was recooled to 0 °C and a solution of chloroacetone (7.48 mL, 91 mmol) in THF (13 mL) was added via syringe pump over 1 hour. Once this addition was complete the reaction mixture was warmed to room temperature and left to stir for 3 hours. The reaction mixture was re-cooled to 0 °C and quenched with water (20 mL) until the solution became clear. The impure reaction mixture was transferred to a 250 mL separating funnel and extracted with diethyl ether (2 × 25 mL). The combined organic extracts were washed with brine (25 mL), dried over magnesium sulphate, filtered and solvent removed under reduced pressure affording a pale yellow liquid. The impure mixture was purified via flash chromatography on silica gel eluting with 5% diethyl ether in petrol. Affording (*Z*)-ethyl 4-chloro-3-methylbut-2-enoate (**59**) and (*E*)-ethyl 4-chloro-3-methylbut-2-

enoate (**58**). Overall isolated yield of pure individual isomers was 71% after 2 columns. There was overlap between the fractions so the yield is higher and has been estimated to be ~90% by ^{1}H NMR.

(*Z*)-ethyl 4-chloro-3-methylbut-2-enoate ((*Z*)-**59**). ^{1}H NMR (500 MHz, $CDCl_3$) δ 5.79 (s, 1H), 4.67 (s, 2H), 4.17 (q, *J*7.1 Hz, 2H), 2.02 (s, 3H), 1.28 (t, *J*7.1 Hz, 3H). ^{13}C NMR (126 MHz, $CDCl_3$) δ 165.42, 152.31, 119.64, 60.20, 42.18, 22.83, 14.21. FT-IR KBr (neat): 2984, 1714, 1651, 1253, 1165,1052 cm^{-1}. m/z LCMS $[ES]^+$ M+Na 186.0

(*E*)-ethyl 4-chloro-3-methylbut-2-enoate ((*E*)-**58**). ^{1}H NMR (500 MHz, $CDCl_3$) δ 6.03 – 5.87 (m, 1H), 4.17 (q, *J*7.1 Hz, 2H), 4.03 (s, 2H), 2.23 (s, 3H), 1.28 (t, *J*7.1 Hz, 3H). ^{13}C NMR (126 MHz, $CDCl_3$) δ 166.02, 151.93, 119.00, 60.09, 49.93, 16.71, 14.25 cm^{-1}. m/z LCMS $[ES]^+$ M+Na 186.0. FT-IR KBr (neat): 2983, 1714, 1655, 1251, 1163,1054 cm^{-1}. The data was in agreement with the literature [48].

Synthesis of (*E*)-4-chloro-3-methylbut-2-en-1-ol ((*E*)-**60**)

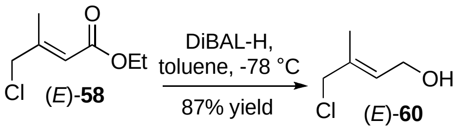

A flame-dried 250 mL round-bottomed flask was charged with (*E*)-ethyl 4-chloro-3-methylbut-2-enoate [(*E*)-**58**, 2.26 g, 13.9 mmol] in toluene (20 mL). The reaction was cooled to -78 °C in a cardice-acetone bath followed by addition of Dibal-H (1.2 M, 25.5 mL, 30.6 mmol) via syringe pump over 1 hour and left to stir at -78 °C for 1 hour. The solution was quenched with dry methanol (5 mL) added dropwise and left to stir for 15 minutes. Rochelles salt (1.2 M, 25 mL) was added and the solution was warmed to room temperature and left stirring vigorously for 6 hours. The solution was transferred to a 250 mL separating funnel and extracted with diethyl ether (2 × 20 mL). The combined organic extracts were washed with brine (20 mL), dried with magnesium sulphate, filtered and the solvent was removed under reduced pressure affording a colourless liquid. Subsequent physiochemical analysis confirmed this was the title product (*E*)-4-chloro-3-methylbut-2-en-1-ol [(E)-**60**, 1.45 g, 12.0 mmol, 87%]. The product was used directly in the next step.

^{1}H NMR (500 MHz, $CDCl_3$) δ 5.74 (t, *J*5.6 Hz, 1H), 4.21 (d, *J*6.2 Hz, 2H), 4.02 (s, 2H), 1.79 (s, 3H). ^{13}C NMR (126 MHz, $CDCl_3$) δ 134.73, 128.87, 59.25, 51.36, 14.46. FT-IR KBr (neat): 3334, 2919, 1445, 1258, 1004 cm^{-1}. m/z LCMS $[ES]^+$ C_5H_8ClKO 157.1. The data was in agreement with the literature [S1].

Synthesis of (*Z*)-4-chloro-3-methylbut-2-en-1-ol ((*Z*)-**61**)

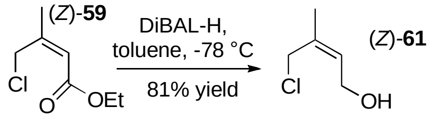

A flame-dried 250 mL round-bottomed flask was charged with (*Z*)-ethyl 4-chloro-3-methylbut-2-enoate [(*Z*)-**59**, 1g, 6.15 mmol] in toluene (20 mL). The reaction was cooled to -78 °C in a cardice-acetone bath followed by addition of Dibal-H (1.2 M, 11.3 mL, 13.5 mmol) via syringe pump over 1 hour and left to stir at -78 °C for 1 hour. The solution was quenched with dry methanol (5 mL) added dropwise and left to stir for 15 minutes. Rochelles salt (1.2 M, 25 mL) was added and the solution was warmed to room temperature and left stirring vigorously for 6 hours. The solution was transferred to a 250 mL separating funnel and extracted with diethyl ether (2 × 20 mL). The combined organic extracts were washed with brine (20 mL), dried with magnesium sulphate, filtered and solvent removed under reduced pressure affording a colourless liquid. Subsequent physiochemical analysis confirmed this was the title product (*Z*)-4-chloro-3-methylbut-2-en-1-ol [(*Z*)-**61**, 600 mg, 5 mmol, 81%] was used directly in the next step.

^{1}H NMR (500 MHz, $CDCl_3$) δ 5.63 (td, *J* 7.0, 1.4 Hz, 1H), 4.22 (dd, *J* 7.0, 0.9 Hz, 2H), 4.09 (s, 2H), 1.88 (d, *J* 1.2 Hz, 3H), 1.53 (s, 1H). ^{13}C NMR (126 MHz, $CDCl_3$) δ 135.43, 129.20, 58.66, 42.88, 21.75. FT-IR KBr (neat): 3334, 2919, 1445, 1258, 1004 cm^{-1}. m/z LCMS [ES]$^+$ C_5H_8ClKO 157.1

Synthesis of (*E*)-4-hydroxy-2-methylbut-2-enyl nitrate ((*E*)-**10**)

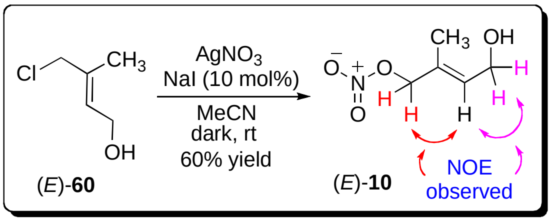

A 20 mL round-bottomed flask was charged with (*E*)-4-chloro-3-methylbut-2-en-1-ol [(*E*)-**60**, 1.4 g, 11.6 mmol] in acetonitrile (5 mL) and wrapped in aluminium foil. To this was added sodium iodide (170 mg, 1.16 mmol) and left to stir for 30 minutes. To this silver nitrate (1.9 g, 11.6 mmol) was added and left to stir in the dark for 16 hours. The solution was filtered through celite and transferred to a 100 mL separating funnel, diluted with water (10 mL) and extracted with diethyl ether (2 × 25 mL). The combined organic extracts were washed with brine (10 mL), dried over magnesium sulphate, filtered and the solvent removed under reduced pressure affording a colourless liquid. The impure reaction mixture was purified via flash chromatography on silica gel eluting with 10% diethyl ether in pentane affording a colourless liquid. Subsequent physiochemical analysis confirmed this to be the title product (*E*)-4-hydroxy-2-methylbut-2-enyl nitrate, (*E*)-**10** (1g, 6.8 mmol, 60%).

^{1}H NMR (500 MHz, $CDCl_3$) δ 5.79 (t, *J* 5.8 Hz, 1H), 4.84 (s, 2H), 4.24 (d, *J* 6.4 Hz, 2H), 1.75 (s, 3H), 1.58 (s, 1H). ^{13}C NMR (126 MHz, $CDCl_3$) δ 131.29, 129.89,

77.89, 58.92, 14.33. FT-IR KBr (neat):3349(OH), 2924(alkene), 1633 and 1280 (ONO_2) cm^{-1}. m/z GCMS $[ES]^-$ 46 NO_2, 62 NO_3, 101 $C_5H_9O_2$.

Synthesis of (*Z*)-4-hydroxy-2-methylbut-2-enyl nitrate ((*Z*)-**9**)

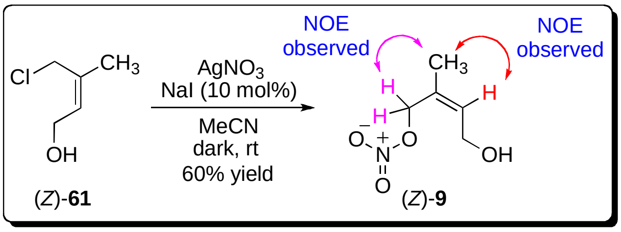

A 20 mL round-bottomed flask was charged with (*Z*)-4-chloro-3-methylbut-2-en-1-ol [(*Z*)-**61**, 2 g, 16.6 mmol] in acetonitrile (10 mL) and wrapped in aluminium foil. To this was added sodium iodide (250 mg, 1.7 mmol) and left to stir for 30 minutes, before silver nitrate (2.82 g, 16.6 mmol) was added and left to stir in the dark for 16 hours. The solution was filtered through celite and transferred to a 100 mL separating funnel, diluted with water (10 mL) and extracted with diethyl ether (2 × 25 mL). The combined organic extracts were washed with brine (10 mL), dried over magnesium sulphate, filtered and solvent removed under reduced pressure affording a colourless liquid. The impure reaction mixture was purified via flash chromatography on silica gel eluting with 10% diethyl ether in pentane affording a colourless liquid. Subsequent physiochemical analysis confirmed this to be the title product (*Z*)-4-hydroxy-2-methylbut-2-enyl nitrate, (*Z*)-**9** (1.45 g, 9.9 mmol, 60%).

^1H NMR (500 MHz, $CDCl_3$) δ 5.78 (t, *J*6.4 Hz, 1H), 4.97 (s, 2H), 4.24 (d, *J*6.7 Hz, 2H), 1.84 (d, *J*1.0 Hz, 3H), 1.60 (s, 1H). ^{13}C NMR (126 MHz, $CDCl_3$) δ 132.09, 130.21, 71.43, 58.58, 21.40. FT-IR KBr (neat):3340 (OH), 2921 (alkene), 1630 and 1277 (ONO_2) cm^{-1}. m/z GCMS $[ES]^-$ 46 NO_2, 62 NO_3, 101 $C_5H_9O_2$.

Synthesis of (*E*)-ethyl 4-(4-methoxybenzyloxy)-3-methylbut-2-enoate ((*E*)-**64**) and (*Z*)-ethyl 4-(4-methoxybenzyloxy)-3-methylbut-2-enoate ((*Z*)-**65**).

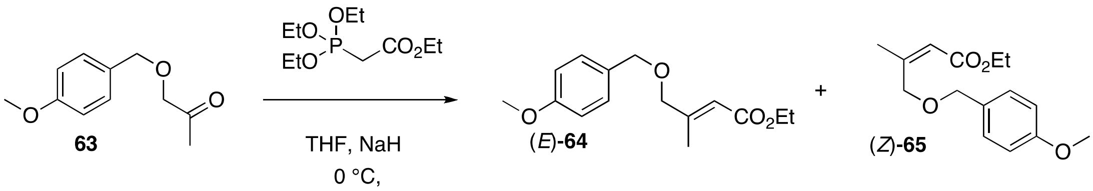

A flame-dried 250 mL round-bottomed flask was charged with THF (50 mL) and cooled to 0 °C in an ice bath. To this sodium hydride (60% in mineral oil, 1.17 g, 29.3 mmol) was added and left to stir for 5 minutes. To this was added triethyl phosphonoacetate (6.46 mL, 32.3 mmol) was added over 1 hour via syringe pump. Following the addition, the solution was allowed to warm to room temperature and left to stir for another 1 hour. The solution was recooled to 0 °C and a solution of **63** (5.7 g, 29.3 mmol) in THF (13 mL) was added via syringe pump over 1 hour. Once the addition was complete the reaction mixture was

warmed to room temperature and left to stir for 3 hours. The reaction mixture was cooled to 0 °C and quenched with water (20 mL) until the solution became clear. The impure reaction mixture was transferred to a 250 mL separating funnel and extracted with diethyl ether (2 x 25 mL). The combined organic extracts were washed with brine (25 mL), dried over magnesium sulphate, filtered and the solvent removed under reduced pressure affording a pale yellow liquid. The impure mixture was purified via flash chromatography on silica gel eluting with 5% ethyl acetate in petrol.

(*E*)-ethyl 4-(4-methoxybenzyloxy)-3-methylbut-2-enoate (**64**) (2.7 g, 10.22 mmol, 35%) ^{1}H NMR (500 MHz, $CDCl_3$) δ 7.27 (d, *J*7.6 Hz, 2H), 6.89 (d, *J*8.2 Hz, 2H), 5.99 (dd, *J*2.9, 1.4 Hz, 1H), 4.46 (s, 2H), 4.17 (q, *J*7.1 Hz, 2H), 3.96 (s, 2H), 3.80 (s, 3H), 2.11 (s, 3H), 1.28 (t, *J*7.1 Hz, 3H). ^{13}C NMR (126 MHz, $CDCl_3$) δ 166.72, 159.34, 154.67, 129.89, 129.33, 115.27, 113.88, 73.85, 72.22, 59.70, 55.29, 15.89, 14.33. FT-IR KBr (neat): 2981, 1715, 1613, 1249, 821 cm^{-1}. m/z LCMS $[ES]^+$ $C_{15}H_{20}KO_4$ 303.0.

(*Z*)-ethyl 4-(4-methoxybenzyloxy)-3-methylbut-2-enoate (**65**) (2 g, 7.57 mmol, 26%) ^{1}H NMR (500 MHz, $CDCl_3$) δ 7.27 (d, *J*8.2 Hz, 2H), 6.87 (d, *J*8.6 Hz, 2H), 5.75 (dd, *J*3.0, 1.5 Hz, 1H), 4.63 (d, *J*0.8 Hz, 2H), 4.44 (s, 2H), 4.13 (q, *J*7.1 Hz, 2H), 3.79 (s, 3H), 1.99 (s, 3H), 1.26 (t, *J*7.1 Hz, 3H). ^{13}C NMR (126 MHz, $CDCl_3$) δ 165.96, 159.22, 157.11, 130.40, 129.30, 117.27, 113.79, 72.38, 69.06, 59.81, 55.27, 21.75, 14.28. FT-IR KBr (neat): 2980, 1713, 1613, 1248, 821 cm^{-1}. m/z LCMS $[ES]^+$ $C_{15}H_{20}NaO_4$ 287.0. The data was in agreement with the literature [S2].

Synthesis of (*E*)-4-(4-methoxybenzyloxy)-3-methylbut-2-en-1-ol ((*E*)-**66**)

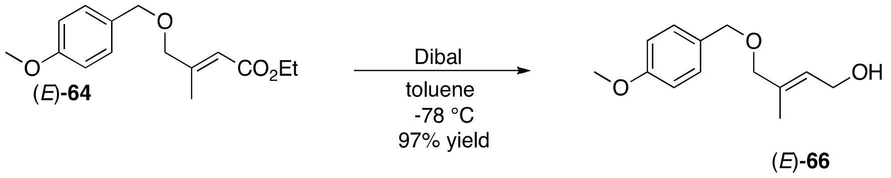

A flame-dried 250 mL round-bottomed flask was charged with (*E*)-ethyl 4-(4-methoxybenzyloxy)-3-methylbut-2-enoate [(*E*)-**64**, 2.2 g, 8.3 mmol] in toluene (20 mL). The reaction was cooled to −78 °C in a cardice-acetone bath followed by addition of Dibal-H (1.2 M, 14.0 mL, 16.7 mmol) via syringe pump over 1 hour and left to stir at −78 °C for 1 hour. The solution was quenched with dry methanol (5 mL) added dropwise and left to stir for 15 minutes. Rochelles salt (1.2 M, 25 mL) was added and the solution was warmed to room temperature and left stirring vigorously for 6 hours. The solution was transferred to a 250 mL separating funnel and extracted with diethyl ether (2 × 20 mL). The combined organic extracts were washed with brine (20 mL), dried with magnesium sulphate, filtered and solvent was removed under reduced pressure affording a colourless liquid. Subsequent physiochemical analysis confirmed this was the title product (*E*)-4-(4-methoxybenzyloxy)-3-methylbut-2-en-1-ol [(*E*)-**66**, 1.8 g, 8.1 mmol, 97%] which was used directly for the next step.

^{1}H NMR (500 MHz, $CDCl_3$) δ 7.26 (d, *J*8.7 Hz, 2H), 6.88 (d, *J*8.7 Hz, 2H), 5.68 (dddd, *J*8.0, 6.7, 2.6, 1.3 Hz, 1H), 4.41 (s, 2H), 4.21 (d, *J*6.7 Hz, 2H), 3.89 (s, 2H),

3.80 (s, 3H), 1.70 (s, 3H), 1.56 (s, 1H). ^{13}C NMR (126 MHz, $CDCl_3$) δ 159.20, 135.77, 130.40, 129.36, 126.12, 113.81, 75.11, 71.66, 59.10, 55.30, 14.07. FT-IR KBr (neat): 3390, 2912, 2856, 1612, 1514, 1248, 1034 cm^{-1}. m/z LCMS $[ES]^+$ $C_{13}H_{18}NaO_3$ 245.1. The data was in agreement with the literature.[47]

Synthesis of (*Z*)-4-(4-methoxybenzyloxy)-3-methylbut-2-en-1-ol ((*Z*)-**67**)

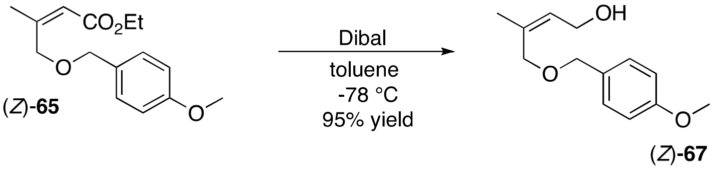

A flame-dried 250 mL round-bottomed flask was charged with (*Z*)-ethyl 4-(4-methoxybenzyloxy)-3-methylbut-2-enoate [(*E*)-**65**, 1.5 g, 5.68 mmol] in toluene (20 mL). The solution was cooled to −78 °C in a cardice-acetone bath followed by the addition of Dibal-H (1.2 M, 10 mL, 12.0 mmol) via syringe pump over 1 hour and left to stir at −78 °C for 1 hour. The solution was quenched with dry methanol (5 mL) added dropwise and left to stir for 15 minutes. Rochelles salt (1.2 M, 25 mL) was added and the solution was warmed to room temperature and left stirring vigorously for 6 hours. The solution was transferred to a 250 mL separating funnel and extracted with diethyl ether (2 × 20 mL). The combined organic extracts were washed with brine (20 mL), dried with magnesium sulphate, filtered and solvent removed under reduced pressure affording a colourless liquid. Subsequent physiochemical analysis confirmed this was the title product (*Z*)-4-(4-methoxybenzyloxy)-3-methylbut-2-en-1-ol [(*Z*)-**67**, 1.8 g, 8.1 mmol, 97%] with no further purification necessary and the product used directly for the next step.

^{1}H NMR (500 MHz, $CDCl_3$) δ 7.26 (d, *J*8.7 Hz, 2H), 6.88 (d, *J*8.7 Hz, 2H), 5.66 (t, *J*7.1 Hz, 1H), 4.42 (s, 2H), 4.11 (d, *J*7.0 Hz, 2H), 4.00 (s, 2H), 3.81 (s, 3H), 1.82 (s, 3H), 1.78 (s, 1H). ^{13}C NMR (126 MHz, $CDCl_3$) δ 159.31, 136.63, 130.07, 129.45, 128.10, 113.87, 71.99, 68.49, 58.68, 55.30, 22.14. FT-IR KBr (neat): 3391, 2914, 2855, 1614, 1515, 1248, 1034 cm^{-1}. m/z LCMS $[ES]^+$ $C_{13}H_{18}NaO_3$ 245.1. The data was in agreement with the literature.[47]

Synthesis of (*E*)-4-(4-methoxybenzyloxy)-3-methylbut-2-enyl nitrate.

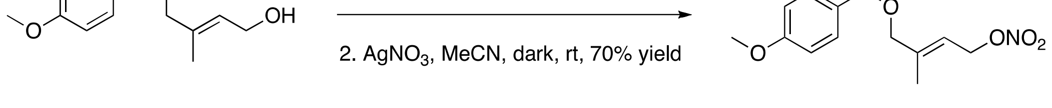

A flame-dried 100 mL round-bottomed flask was charged with (*E*)-4-(4-methoxybenzyloxy)-3-methylbut-2-en-1-ol (1.8 g, 8.1 mmol) in diethyl ether (20 mL) was cooled to 0 °C in an ice bath. To this was added phosphorus tribromide (384 µL, 4.05 mmol) dropwise over 5 minutes. The solution was warmed to ambient temperature and left to stir for 45 minutes, where upon it was recooled to 0 °C and quenched with brine (5 mL) and diluted with water (20 mL). The reaction mixture was transferred to a 100 mL separating funnel and extracted with diethyl

ether (2 × 25 mL). The combined organic extracts were washed with brine (20 mL), dried over magnesium sulphate, filtered and the solvent removed under reduced pressure affording a colourless liquid (which turned brown upon standing) (2.2 g, 95%). The liquid was re-dissolved in acetonitrile (10 mL), the flask wrapped in aluminium foil. To this was added silver nitrate 1.31 g in one portion and left to stir for 16 hours in the dark. The solution was filtered through celite and transferred to a 100 mL separating funnel and diluted with water. The solution was extracted with diethyl ether (2 × 25 mL) and the combined organic extracts were washed with brine (10 mL), dried over magnesium sulphate, filtered and the solvent removed under reduced pressure affording a pale yellow liquid. The impure reaction mixture was purified via flash chromatography eluting with 10% diethyl ether in pentane affording a colourless liquid. Subsequent physiochemical analysis confirmed this was the title product (*E*)-4-(4-methoxybenzyloxy)-3-methylbut-2-enyl nitrate (1.45 g, 5.4 mmol, 70%)

^{1}H NMR (500 MHz, $CDCl_3$) δ 7.26 (d, *J* 8.7 Hz, 2H), 6.89 (d, *J* 8.7 Hz, 2H), 5.71 – 5.59 (m, 1H), 4.99 (d, *J* 7.2 Hz, 2H), 4.43 (s, 2H), 3.92 (s, 2H), 3.81 (s, 3H), 1.77 (s, 3H). ^{13}C NMR (126 MHz, $CDCl_3$) δ 159.30, 142.91, 130.01, 129.40, 115.94, 113.85, 74.05, 71.99, 69.38, 55.31, 14.29. FT-IR KBr (neat):2838, 1628, 1513, 1278, 1248 cm^{-1}. m/z LCMS [ES]$^-$ $C_{13}H_{17}O_3$ 221.1, NO_2 45.9.

Synthesis of (*Z*)-4-(4-methoxybenzyloxy)-3-methylbut-2-enyl nitrate.

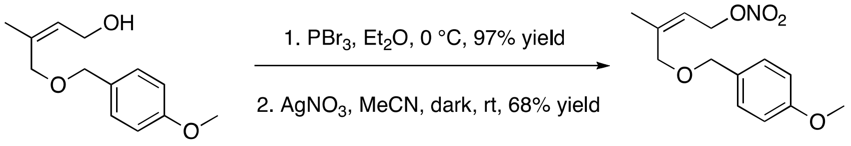

A flame-dried 100 mL round-bottomed flask was charged with (*Z*)-4-(4-methoxybenzyloxy)-3-methylbut-2-en-1-ol (1.2 g, 5.4 mmol) in diethyl ether (20 mL) was cooled to 0 °C in an ice bath. To this was added phosphorus tribromide (256 µL, 4.05 mmol) dropwise over 5 minutes. The solution was warmed to ambient temperature and left to stir for 45 minutes. The solution was recooled to 0 °C, quenched with brine (5 mL) and diluted with water (20 mL). The reaction mixture was transferred to a 100 mL separating funnel and extracted with diethyl ether (2 × 25 mL). The combined organic extracts were washed with brine (20 mL), dried over magnesium sulphate, filtered and solvent removed under reduced pressure affording a colourless liquid (1.5 g, 97%). The liquid was re-dissolved in acetonitrile (10 mL) and the flask wrapped in aluminium foil. To this was added silver nitrate (894 mg, 5.26 mmol) in one portion and left to stir for 16 hours in the dark. The solution was filtered through celite and transferred to a 100 mL separating funnel and diluted with water. The solution was extracted with diethyl ether (2 × 25 mL) and the combined organic extracts were washed with brine (10 mL), dried over magnesium sulphate, filtered and the solvent removed under reduced pressure affording a pale yellow liquid. The impure reaction mixture was purified via flash chromatography eluting with 10% diethyl ether in pentane affording a colourless liquid. Subsequent physiochemical analysis confirmed this was the title product (*Z*)-4-(4-methoxybenzyloxy)-3-methylbut-2-enyl nitrate (960 mg, 3.6 mmol, 68%).

^{1}H NMR (500 MHz, $CDCl_3$) δ 7.26 (d, *J*8.7 Hz, 2H), 6.89 (d, *J*8.7 Hz, 2H), 5.65 (ddd, *J*8.7, 5.9, 1.4 Hz, 1H), 4.99 (d, *J*7.3 Hz, 2H), 4.43 (s, 2H), 3.92 (s, 2H), 3.81 (s, 3H), 1.77 (s, 3H). ^{13}C NMR (126 MHz, $CDCl_3$) δ 159.37, 143.01, 129.86, 129.41, 118.41, 113.89, 72.07, 69.13, 68.13, 55.32, 21.96. FT-IR KBr (neat): 2839, 1633, 1514, 1278, 1248, 864 cm^{-1}. m/z LCMS $[ES]^-$ $C_{13}H_{17}O_3$ 221.1, NO_2 45.9.

Synthesis of (*E*)-4-hydroxy-3-methylbut-2-enyl nitrate ((*E*)-**11**)

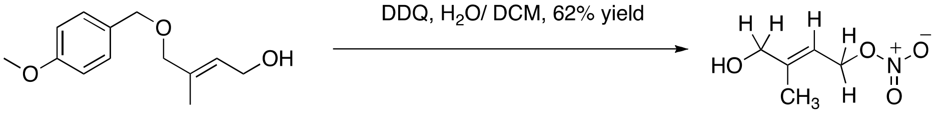

A round-bottomed flask was charged with (*E*)-4-(4-methoxybenzyloxy)-3-methylbut-2-enyl nitrate (960 mg, 3.59 mmol) in DCM (5 mL). To this was added water (7 µL, 0.36 mmol) and the reaction mixture cooled to 0 °C in an ice bath and left to stir for 5 minutes. DDQ (978 mg, 2.21 mmol) was added in 3 portions and left to stir for 30 minutes at 0 °C before removal of the ice bath. The solution was left to stir for 3 hours before being quenched with saturated sodium bicarbonate solution (5 mL). The reaction mixture was transferred to a 100 mL separating funnel and extracted with DCM (2 × 10 mL). The combined organic extracts were washed with brine (10 mL), dried over magnesium sulphate, filtered and the solvent removed under reduced pressure affording a red liquid. The impure reaction mixture was purified via flash chromatography on silica gel eluting with 10% diethyl ether in pentane affording a colourless liquid. Subsequent physiochemical analysis confirmed this to be the titled product (*E*)-4-hydroxy-3-methylbut-2-enyl nitrate [(*E*)-**11**, 325 mg, 2.21 mmol, 62%].

^{1}H NMR (500 MHz, $CDCl_3$) δ 5.63 (t, *J*6.5 Hz, 1H), 4.99 (d, *J*7.2 Hz, 2H), 4.06 (s, 2H), 2.11 (s, 1H), 1.75 (s, 3H). ^{13}C NMR (126 MHz, $CDCl_3$) δ 145.3, 113.9, 69.3, 67.0, 13.9. FT-IR KBr (neat) : 3353 (OH), 2920 (ene), 1633 and 1279 (ONO_2) cm^{-1}. m/z GCMS $[ES]^-$ 46 NO_2, 62 NO_3, 101 $C_5H_9O_2$.

Synthesis of (*Z*)-4-hydroxy-3-methylbut-2-enyl nitrate ((*Z*)-**12**)

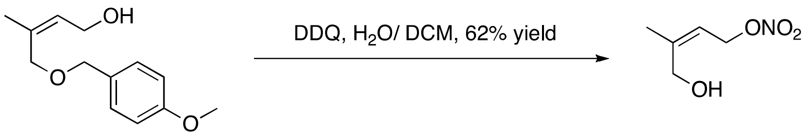

A round-bottomed flask was charged with (*Z*)-4-(4-methoxybenzyloxy)-3-methylbut-2-enyl nitrate (500 mg, 1.87 mmol) in DCM (5 mL). To this was added water (4 µL, 0.19 mmol) was added and the reaction mixture cooled to 0 °C in an ice bath and left to stir for 5 minutes. DDQ (510 mg, 2.25 mmol) was added in 3 portions and left to stir for 30 minutes at 0 °C before removal of the ice bath. The solution was left to stir for 3 hours before being quenched with saturated sodium bicarbonate solution (5 mL). The reaction mixture was transferred to a 100 mL separating funnel and extracted with DCM (2 × 10 mL). The combined organic extracts were washed with brine (10 mL), dried over magnesium sulphate, filtered

and the solvent removed under reduced pressure affording a red liquid. The impure reaction mixture was purified via flash chromatography on silica gel eluting with 10% diethyl ether in pentane affording a colourless liquid. Subsequent physiochemical analysis confirmed this to be the titled product (*Z*)-4-hydroxy-3-methylbut-2-enyl nitrate [(*Z*)-**12**, 145 mg, 0.99 mmol, 53%].

^{1}H NMR (500 MHz, $CDCl_3$) δ 5.47 (t, *J* 7.4 Hz, 1H), 5.00 (d, *J* 7.3 Hz, 2H), 4.23 (s, 2H), 2.96 (s, 1H), 1.87 (s, 3H). ^{13}C NMR (126 MHz, $CDCl_3$) δ 145.07, 117.56, 68.87, 61.60, 21.41. FT-IR KBr (neat): 3348 (OH), 2920 (ene), 1634 and 1279 (ONO_2). m/z GCMS $[ES]^-$ 46 NO_2, 62 NO_3, 99 $C_5H_7O_2$

Synthesis of 1-(4-methoxybenzyloxy)-2-methylbut-3-en-2-ol (*rac*-**68**)

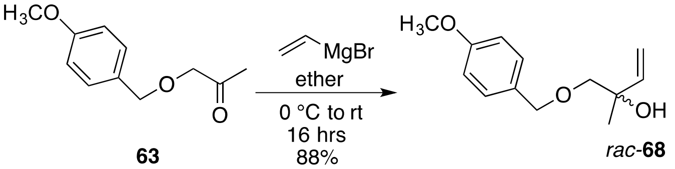

A flame-dried 25 mL round-bottomed flask was charged with **63** (450 mg, 2.3 mmol) in diethyl ether (2 mL). The solution was cooled to 0 °C in an ice bath. To this vinyl magnesium bromide (1 M in THF) (9.3 mL, 9.3 mmol) was added dropwise via syringe. The resulting solution was left to warm to ambient temperature and left to stir for 16 hours. The impure reaction mixture was filtered through celite and the solvent removed under reduced pressure.The colourless oil was purified via flash chromatography on silica gel eluting with 20% diethyl ether in pentane affording a colourless oil. Subsequent physiochemical analysis confirmed this was the title product, *rac*-**68** (453 mg, 2.0 mmol, 88%).

^{1}H NMR (500 MHz, $CDCl_3$) δ 7.25 (d, *J* 8.7 Hz, 2H), 6.88 (d, *J* 8.7 Hz, 2H), 5.91 (dd, *J* 17.3, 10.8 Hz, 1H), 5.31 (dd, *J* 17.3, 1.3 Hz, 1H), 5.12 (dd, *J* 10.8, 1.3 Hz, 1H), 4.51 (s, 2H), 3.81 (s, 3H), 3.34 (dd, *J* 23.5, 9.0 Hz, 2H), 2.47 (s, 1H), 1.26 (s, 3H). ^{13}C NMR (126 MHz, $CDCl_3$) δ 159.29, 142.47, 130.09, 129.30, 113.84, 113.33, 76.93, 73.18, 72.70, 55.29, 24.45. FT-IR KBr (neat) : 3460, 2859, 1613, 1514, 1462, 1248, 1092 cm^{-1}. m/z LCMS $[ES]^+$ $C_{13}H_{18}NaO_3$: 245.1. The data was in agreement with the literature [S3].

Synthesis of *tert*-butyl(1-(4-methoxybenzyloxy)-2-methylbut-3-en-2-yloxy)dimethylsilane (*rac*-**69**)

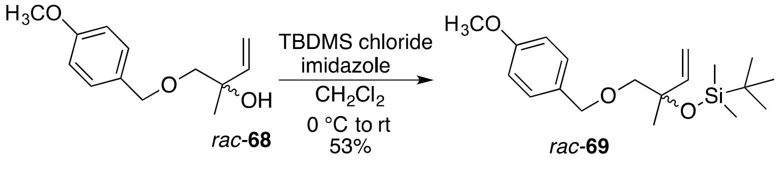

A flame-dried 25 mL round-bottomed flask was charged with 1-(4-methoxybenzyloxy)-2-methylbut-3-en-2-ol, (*rac*-**68**, 125 mg, 0.56 mmol) and imidazole (96 mg, 1.4 mmol) in dichloromethane. The solution was cooled to 0 °C in an ice bath. TBDMS Cl (102 mg, 0.68 mmol) was added and the solution was warmed to ambient temperature and left to stir for 24 hours. After 24 hours the

solution was absorbed onto silica gel and purified via flash chromatograpy on silica gel eluting with 10% diethyl ether in petrol affording a colourless liquid. Subsequent physiochemical analysis confirmed this to be the titled product, *rac*-**69** (101 mg, 0.3 mmol, 53%).

^{1}H NMR (500 MHz, $CDCl_3$) δ 7.25 (d, *J*9.0 Hz, 2H), 6.87 (d, *J*8.7 Hz, 2H), 5.94 (dd, *J*17.3, 10.7 Hz, 1H), 5.26 (d, *J*15.7 Hz, 1H), 5.06 (d, *J*12.3 Hz, 1H), 4.48 (s, 2H), 3.81 (s, 3H), 3.29 (s, 2H), 1.33 (s, 3H), 0.88 (s, 9H), 0.07 (s, 6H). ^{13}C NMR (126 MHz, $CDCl_3$) δ 161.20, 145.82, 132.93, 131.21, 115.83, 115.17, 80.49, 77.74, 75.27, 57.43, 28.07, 26.86, 20.45, 3.20, -0.00. FT-IR KBr (neat) : 2956, 2930, 1614, 1514, 1249, 1039 cm^{-1}. m/z LCMS $[ES]^+$ $C_{19}H_{32}NaO_3Si$ 359.2. HRMS (NESP) m/z: $[M + NH_4]^+$ Calcd for $C_{19}H_{36}NO_3Si$ 354.2464; Found 354.2467.

Synthesis of 2-(*tert*-butyldimethylsilyloxy)-2-methylbut-3-en-1-ol (*rac*-**70**)

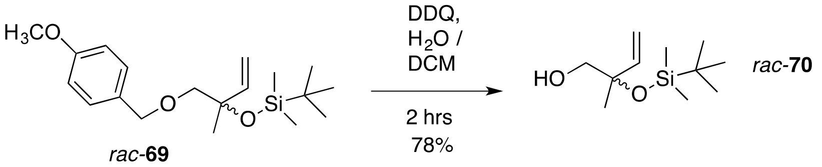

A 25 mL round-bottomed flask was charged with *tert*-butyl(1-(4-methoxybenzyloxy)-2-methylbut-3-en-2-yloxy)dimethylsilane (*rac*-**69**, 30 mg, 0.09 mmol) in DCM (2 mL). To this was added one drop of water was added and the reaction cooled to 0 °C in an ice bath. DDQ (22 mg, 0.1 mmol) was added in portions and left to stir for 10 minutes at 0 °C before being warmed to ambient temperature and left to stir for 2 hours. The reaction mixture was quenched with saturated sodium bicarbonate solution (1 mL) and transferred to a separating funnel and the aqueous layer was extracted with DCM (2 x 3 mL), washed with brine (3 mL), dried over magnesium sulphate, filtered and the solvent removed under reduced pressure. The impure material was purified via flash chromatography on silica gel eluting with 10% diethyl ether in petrol affording *rac*-**70** as a colourless liquid. Subsequent physiochemical analysis confirmed this was the titled product (15 mg, 0.07 mmol, 78%).

^{1}H NMR (500 MHz, $CDCl_3$) δ 5.90 (dd, *J*17.5, 10.8 Hz, 1H), 5.24 (dd, *J*17.5, 1.3 Hz, 1H), 5.14 (dd, *J*10.8, 1.3 Hz, 1H), 3.36 (ddd, *J*26.2, 10.6, 6.5 Hz, 2H), 1.96 (t, *J*6.5 Hz, 1H), 1.33 (s, 3H), 0.89 (s, 9H), 0.10 (d, *J*7.0 Hz, 6H). ^{13}C NMR (126 MHz, $CDCl_3$) δ 142.53, 114.42, 76.20, 71.08, 25.84, 23.30, 18.19, -2.28 (d, *J*10.6 Hz). FT-IR KBr (neat) : 3462, 2956, 2930, 2858, 1472, 1254, 1056 cm^{-1}. m/z LCMS $[ES]^+$ $C_{11}H_{24}NaO_2Si$ 239.1. HRMS (NESP) m/z: $[M + NH_4]^+$ Calcd for $C_{11}H_{28}NO_2Si$ 234.1889; Found 234.1887.

Synthesis of 2-(*tert*-butyldimethylsilyloxy)-2-methylbut-3-enyl methanesulfonate (*rac*-**71**)

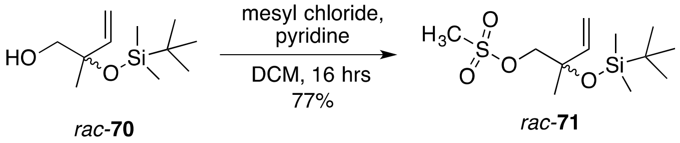

A flame-dried 25 mL round-bottomed flask was charged with 2-(*tert*-butyldimethylsilyloxy)-2-methylbut-3-en-1-ol (*rac*-**70**, 20 mg, 0.1 mmol), pyridine (22 µL, 0.28 mmol) in DCM (1 mL). The resulting solution was cooled to 0 °C in an ice bath before addition of methanesulfonyl chloride (8 µL, 0.1 mmol). The solution was left to stir at 0 °C for 10 minutes before being warmed to ambient temperature and was left to stir for 4 hours. The impure reaction mixture was absorbed onto silica gel and purified via flash chromatography on silica gel eluting with 10% diethyl ether in petrol affording a colourless oil. Subsequent physiochemical analysis confirmed this to be the title compound, *rac*-**71** (21 mg, 0.07 mmol, 77%).

^{1}H NMR (500 MHz, $CDCl_3$) δ 5.88 (dd, *J*17.4, 10.7 Hz, 1H), 5.32 (dd, *J*17.4, 1.0 Hz, 1H), 5.19 (dd, *J*10.7, 1.0 Hz, 1H), 3.97 (s, 2H), 3.00 (s, 3H), 1.40 (s, 3H), 0.88 (s, 9H), 0.10 (d, *J*8.4 Hz, 6H). ^{13}C NMR (126 MHz, $CDCl_3$) δ 143.29, 117.69, 77.94, 76.16, 39.59, 27.92, 25.94, 20.36, -0.04 (d, *J*9.9 Hz). FT-IR KBr (neat) : 2932, 1356, 1253, 1174, 961, 833 cm^{-1}. m/z LCMS $[ES]^+$ $C_{12}H_{26}NaO_4SSi$ 317.1. HRMS (NESP) m/z: $[M + Na]^+$ Calcd for $C_{12}H_{26}NaO_4SSi$ 317.1219; Found 317.1217.

Synthesis of 3-(bromomethyl)-2,2-dimethyloxirane (*rac*-**74**)

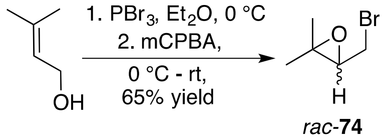

A flame-dried round-bottomed flask was charged with 3-methyl-2-buten-1-ol (1 g, 11.61 mmol) in diethyl ether (5 mL) and cooled to 0 °C in an ice bath. To this was added phosphorous tribromide (518 µL, 5.46 mmol) dropwise and left to stir for 30 minutes. The reaction mixture was quenched with brine (5 mL) and left to stir for 10 minutes. The solution was transferred to a 25 mL separating funnel and extracted with diethyl ether (2 × 5 mL). The combined organic extracts were washed with brine (5 mL), dried over magnesium sulphate, filtered and the solvent removed under reduced pressure. The resulting liquid was redissolved in dichloromethane (10 mL) and cooled to 0 °C in an ice bath. To this was added mCPBA 70% (4.0 g, 17.4 mmol) was added in 2 equal portions and stirred at 0 °C for 30 minutes before warming to room temperature and leaving to stir for 4 hours. The precipitate was filtered off and an aqueous solution of sodium hydrosulfite (5%, 5 mL) was added to the filtrate and left to stir for 1 hour. The biphasic solution was transferred to a 25 mL separating funnel and extracted with dichloromethane (2 × 5mL). The combined organic extracts were washed with saturated sodium bicarbonate solution (10 mL), brine, dried over magnesium

sulphate, filtered and solvent removed under reduced pressure. The resulting pale yellow liquid was purified via flash chromatography on silica eluting with 2% diethyl ether in pentane to afford a colourless liquid. Subsequent physiochemical analysis confirmed this was *rac*-**74** (1.25 g, 7.57 mmol, 65%).

^{1}H NMR (500 MHz, $CDCl_3$) δ 3.50 (dd, *J*10.5, 6.1 Hz, 1H), 3.24 (dd, *J*10.5, 7.6 Hz, 1H), 3.07 (dd, *J*7.5, 6.1 Hz, 1H), 1.34 (s, 3H), 1.31 (s, 3H). ^{13}C NMR (126 MHz, $CDCl_3$) δ 62.22, 60.68, 29.89, 24.48, 18.22. The data was in agreement with the literature [S4].

Synthesis of 1-bromo-3-methylbut-3-en-2-yl acetate (*rac*-**76**)

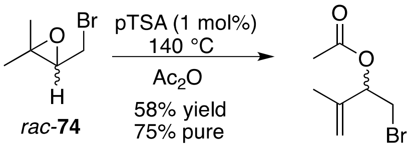

A two-necked round-bottomed flask was charged with acetic anhydride (9.8 mL, 104 mmol) and *p*-toluenesulfonic acid monohydrate (197 mg, 1.04 mmol). The solution was heated to reflux ~140 °C with vigorous stirring. 3-(Bromomethyl)-2,2-dimethyloxirane (*rac*-**74**, 1.7 g, 10.37 mmol) was added quickly via syringe to the refluxing mixture and left to reflux for 15 minutes before being cooled to room temperature. The mixture was transferred to a 50 mL separating funnel and extracted with diethyl ether (2 × 5 mL). The combined extracts were washed with brine (10 mL), dried over magnesium sulphate, filtered and solvent removed under reduced pressure. The impure reaction mixture was purified via flash chromatography on silica gel eluting with 2% diethyl ether in pentane. Subsequent physiochemical analysis afforded an inseparable mixture of *rac*-**76** and a by-product. This was estimated to be ~75% pure by ^{1}H-NMR. This was used directly for the next step. The data was in agreement with the literature [S5].

Synthesis of (±)-3-methyl-2-(nitrooxy)but-3-enyl acetate (*rac*-**83**)

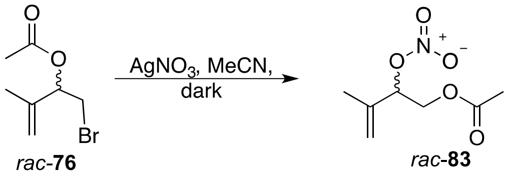

A 25 mL round-bottomed flask, wrapped in aluminium foil, was charged with 1-bromo-3-methylbut-3-en-2-yl acetate (*rac*-**76**, 75%, 310 mg, 1.5 mmol) in acetonitrile (5 mL). To this was added silver nitrate (254 mg, 1.5 mmol) was added in one portion and left to stir for 16 hours. The precipitate was filtered off and the filtrate transferred to a 25 mL separating funnel and extracted with diethyl ether (2 × 5 mL). The combined organic extracts were dried with magnesium sulphate, filtered and the solvent removed under reduced pressure. The reaction mixture was purified via flash chromatography on silica gel eluting with 10% diethyl ether in pentane affording *rac*-**83** as a colourless oil. Subsequent

physiochemical analysis confirmed this was the title product (198 mg, 1.05 mmol, 70%).

^{1}H NMR (500 MHz, $CDCl_3$) δ 5.44 (dd, *J* 8.3, 3.1 Hz, 1H), 5.16 (t, *J* 1.3 Hz, 1H), 5.11 (s, 1H), 4.31 (dd, *J* 12.5, 3.2 Hz, 1H), 4.17 (dd, *J* 12.5, 8.3 Hz, 1H), 2.09 (s, 3H), 1.83 (s, 3H). ^{13}C NMR (126 MHz, $CDCl_3$) δ 170.53, 137.92, 115.89, 83.29, 62.16, 20.71, 19.06. FT-IR KBr (neat) : 2958, 1748, 1638, 1277, 1222, 855 cm^{-1}. m/z LCMS $[ES]^+$ $C_5H_8KNO_4$ 130.1, NO_2 45.0

Synthesis of 2-hydroxy-3-methylbut-3-enyl nitrate (*rac*-**16**)

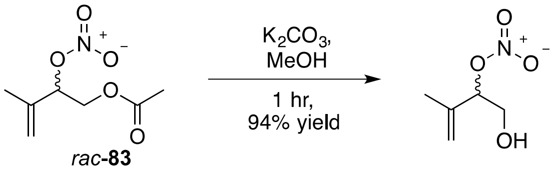

rac-**83**

rac-**16**
isolated

A 10 mL round-bottomed flask was charged with (±)-3-methyl-2-(nitrooxy)but-3-enyl acetate (*rac*-**83**, 30 mg, 0.16 mmol) in methanol (2 mL). To this was added potassium carbonate (44 mg, 0.32 mmol) in one portion and left to stir at ambient temperature for 1 hour. The reaction mixture was transferred to a 25 mL separating funnel, diluted with water (5 mL) and extracted with diethyl ether (2 × 2 mL). The combined organic extracts were washed with brine (5 mL), dried over magnesium sulphate, filtered and the solvent removed under reduced pressure affording a colourless liquid. The reaction mixture was purified via flash chromatography on silica eluting with 15% diethyl ether in pentane affording a colourless liquid. Subsequent physiochemical analysis confirmed this to be *rac*-**16** (22 mg, 0.15 mmol, 94%).

^{1}H NMR (500 MHz, $CDCl_3$) δ 5.33 (t, *J* 5.7 Hz, 1H), 5.14 (dd, *J* 1.8, 0.9 Hz, 1H), 5.11 – 5.08 (m, 1H), 3.80 (d, *J* 6.0 Hz, 2H), 1.81 (s, 3H), 1.57 (s, 1H). ^{13}C NMR (126 MHz, $CDCl_3$) δ 138.42, 115.22, 86.65, 61.71, 19.19. FT-IR KBr (neat) : 3359, 1634, 1274, 856 cm^{-1}. m/z GCMS $[ES]^-$ 46 NO_2, 101 $C_5H_9O_2$.

Synthesis of (1*R*, 5*S*)-(6,6-dimethylbicyclo[3.1.1]hept-2-en-2-yl)methyl nitrate (**86**)

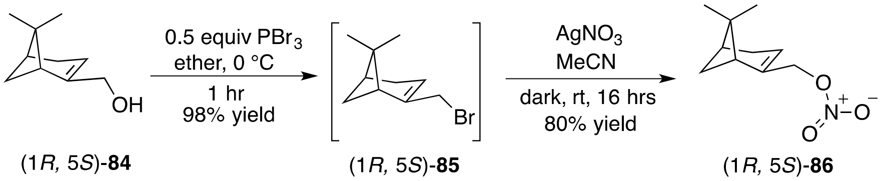

(1*R*, 5*S*)-**84** (1*R*, 5*S*)-**85** (1*R*, 5*S*)-**86**

A flame-dried 25 mL round-bottomed flask was charged with myrtenol (500 mg, 3.28 mmol) in diethyl ether (5 mL) and cooled to 0 °C in an ice bath. To this was added phosphorous tribromide (156 µL, 1.64 mmol) dropwise and left to stir at 0 °C for 1 hour. The impure reaction mixture was quenched with brine (5 mL) and transferred to a 25 mL separating funnel. The solution was extracted with diethyl ether (2 × 10 mL) and the combined organic extracts were washed with brine (5 mL), dried over magnesium

sulphate, filtered and the solvent removed under reduced pressure affording a colourless oil (692 mg, 3.28 mmol, 98%, the oil turned brown after 5 minutes and was used directly for the next step). The impure (1*R*, 5*S*)-2-(bromomethyl)-6,6-dimethylbicyclo[3.1.1]hept-2-ene (200 mg, 0.93 mmol) was dissolved in acetonitrile (2 mL) and the flask wrapped in aluminium foil. To this silver nitrate (158 mg, 0.93 mmol) was added in one portion and left to stir in the dark for 12 hours. The silver bromide precipitate was filtered off and washed with diethyl ether (2 × 10 mL). The filtrate was transferred to a 25 mL separating funnel and diluted with water (5 mL) before being extracted with diethyl ether (2 × 5 mL). The combined organic extracts were dried over magnesium sulphate, filtered and the solvent removed under reduced pressure affording a pale yellow oil. The impure reaction mixture was purified via flash chromatography on silica gel, eluting with 10% diethyl ether in pentane affording (1*R*,5*S*)-**86** colourless oil. Subsequent physiochemical analysis confirmed this to be the title product (147 mg, 0.75 mmol, 80%).

$[\alpha]_D^{24}$ -55 (c 1.0, $CHCl_3$), ^{1}H NMR (500 MHz, $CDCl_3$) δ 5.76 (ddd, *J*4.3, 2.9, 1.4 Hz, 1H), 4.80 (qd, *J*12.1, 1.2 Hz, 2H), 2.43 (dt, *J*8.8, 5.6 Hz, 1H), 2.31 (q, *J*18.4 Hz, 2H), 2.19 (td, *J*5.6, 1.5 Hz, 1H), 2.12 (tdd, *J*4.2, 2.9, 1.3 Hz, 1H), 1.30 (s, 3H), 1.16 (d, *J*8.8 Hz, 1H), 0.81 (s, 3H). ^{13}C NMR (126 MHz, $CDCl_3$) δ 139.79, 126.43, 76.12, 43.76, 40.38, 38.11, 31.55, 25.97, 20.96. FT-IR KBr(neat): 2935, 1630, 1277, 856 cm^{-1}. m/z GCMS $[ES]^-$ 46 NO_2, 149 $C_{10}H_{13}O$.

Title: ^{1}H and ^{13}C NMR spectra of the core starting materials and IPNs

^{1}H NMR and ^{13}C NMR of (*E*)-**58**

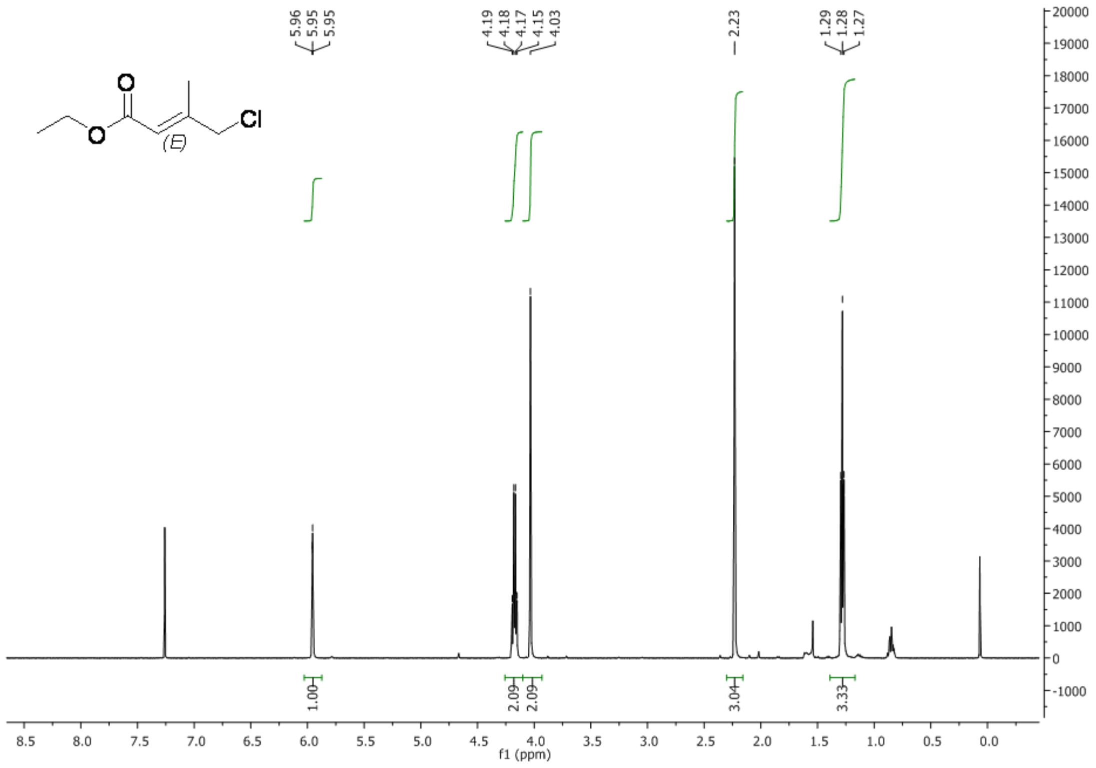

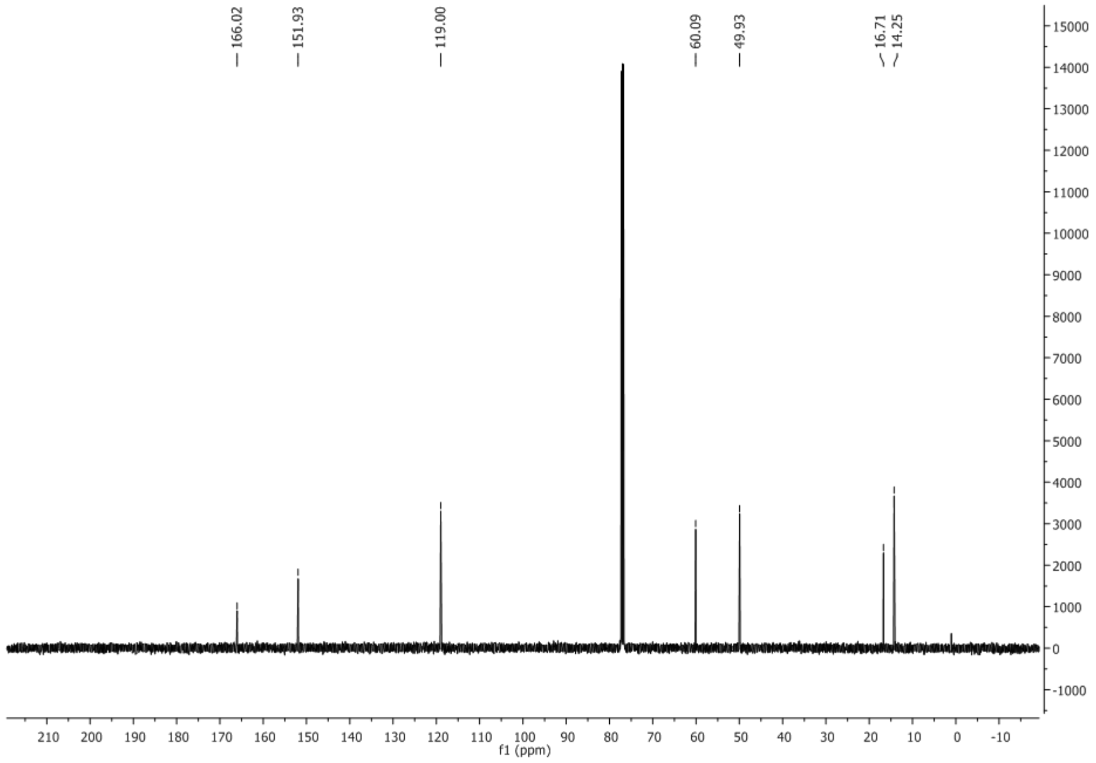

^{1}H NMR and ^{13}C NMR of (*Z*)-**59**

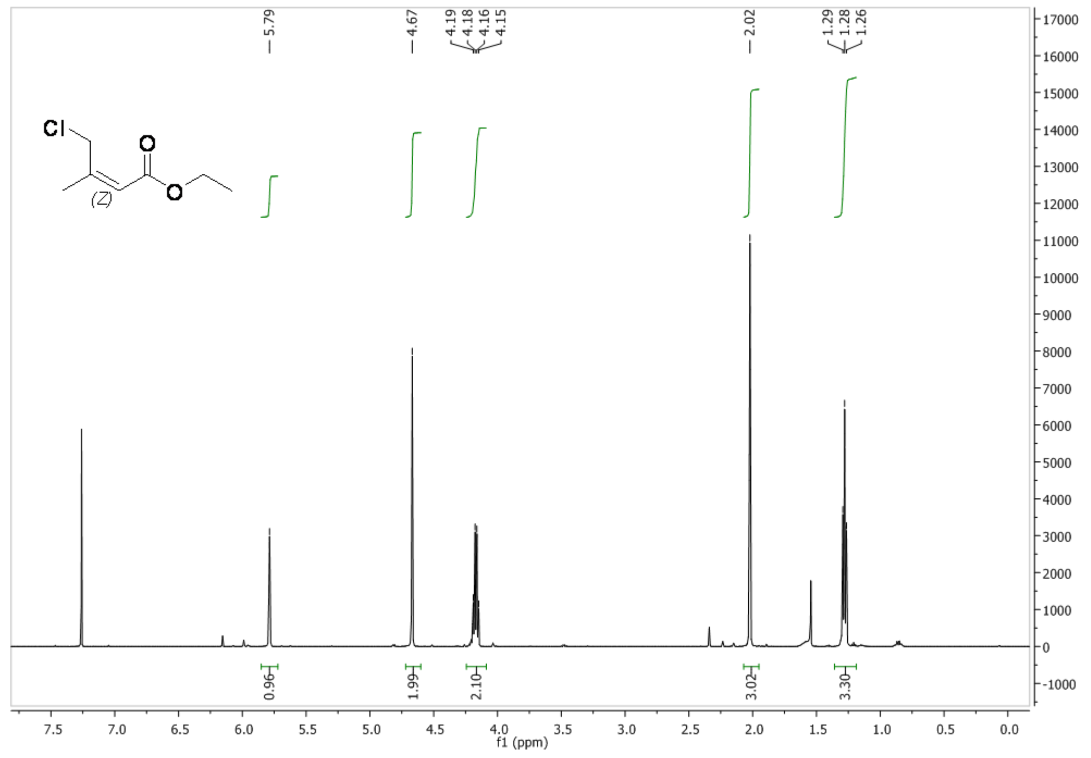

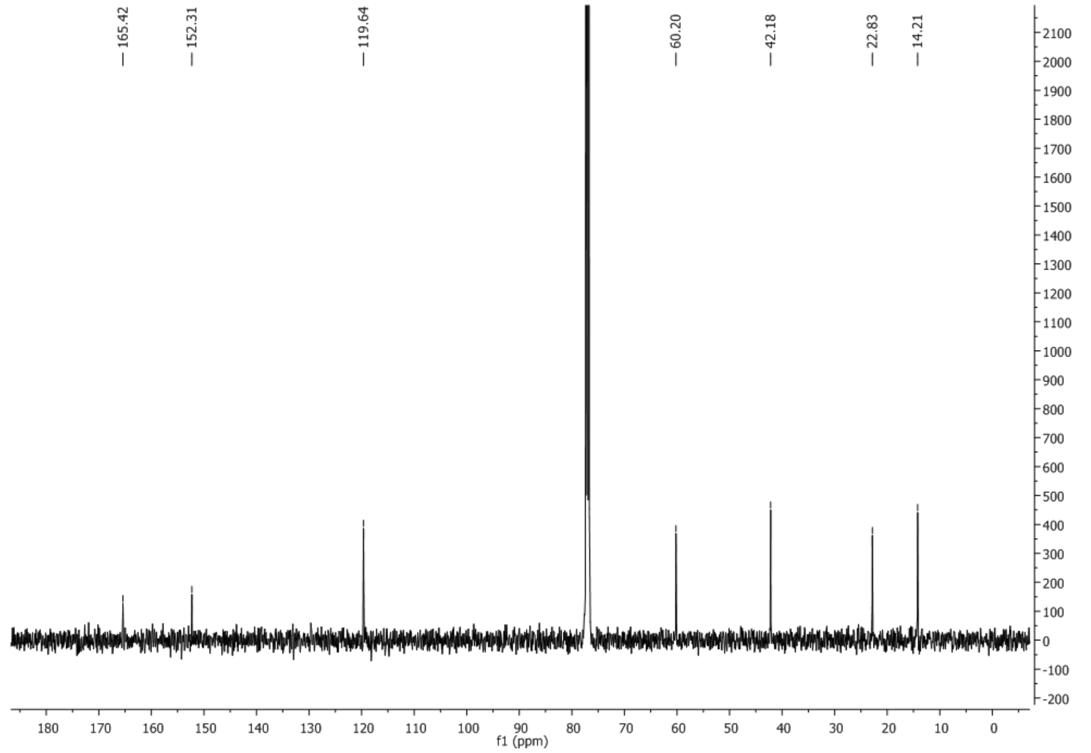

^{1}H NMR and ^{13}C NMR of (*E*)-**60**

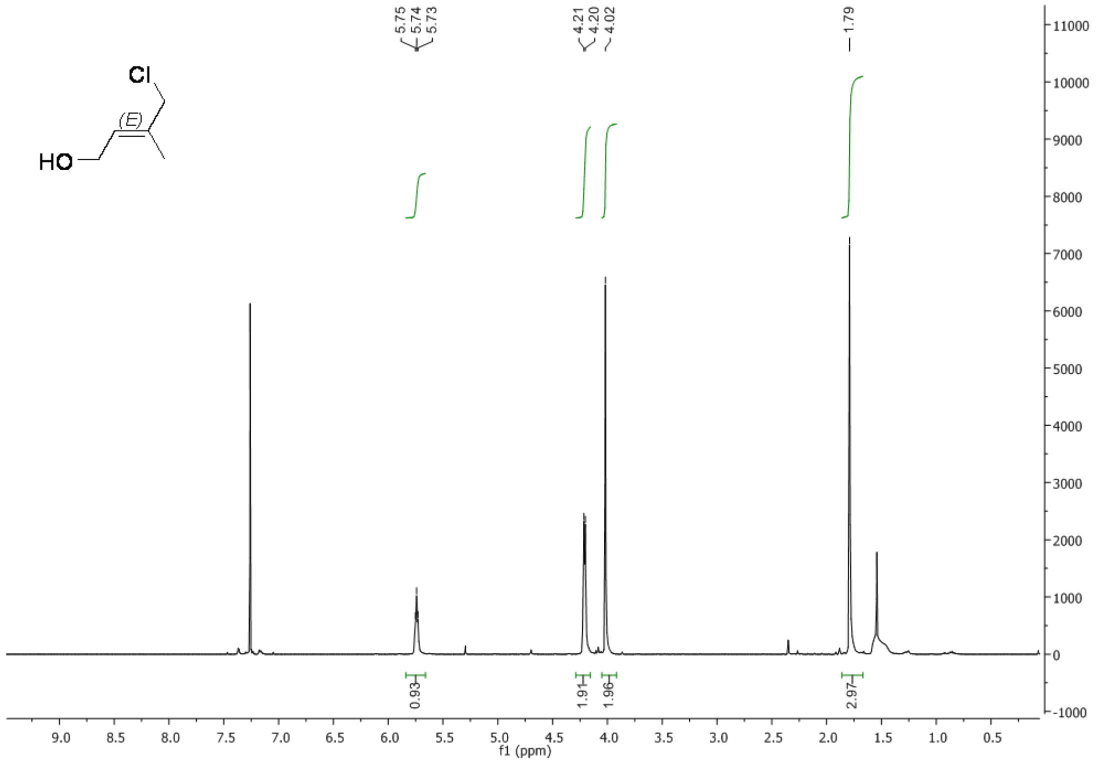

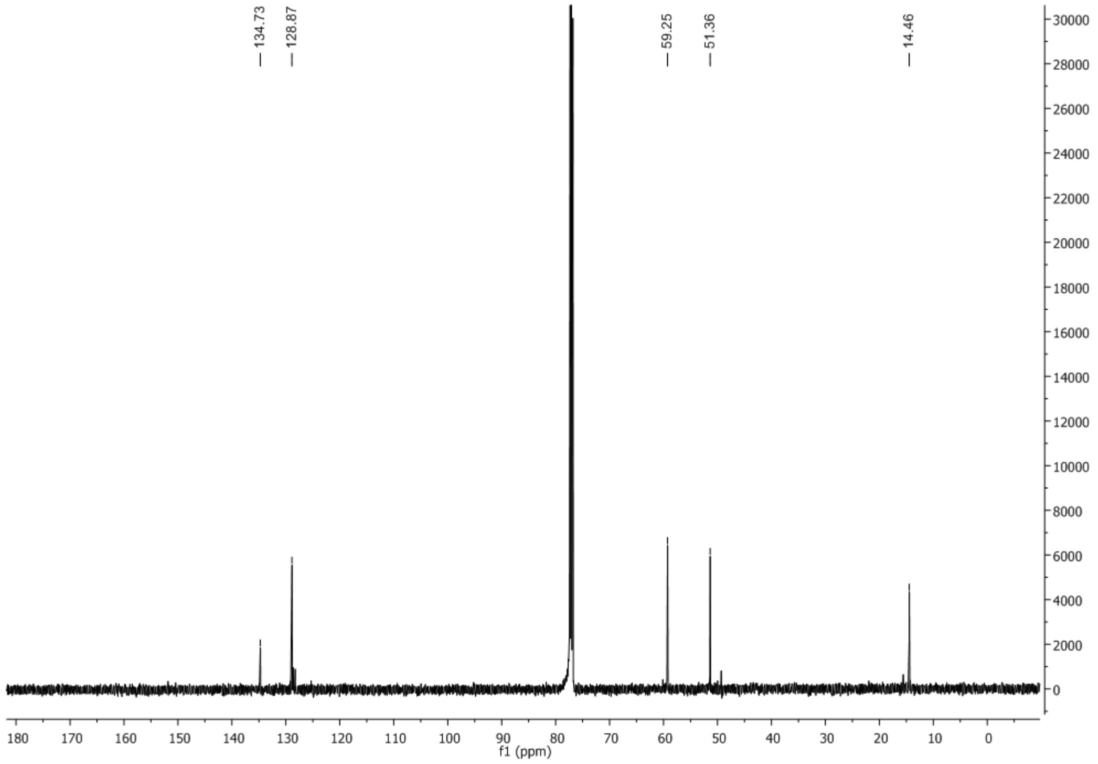

^{1}H NMR and ^{13}C NMR of (E)-**10**

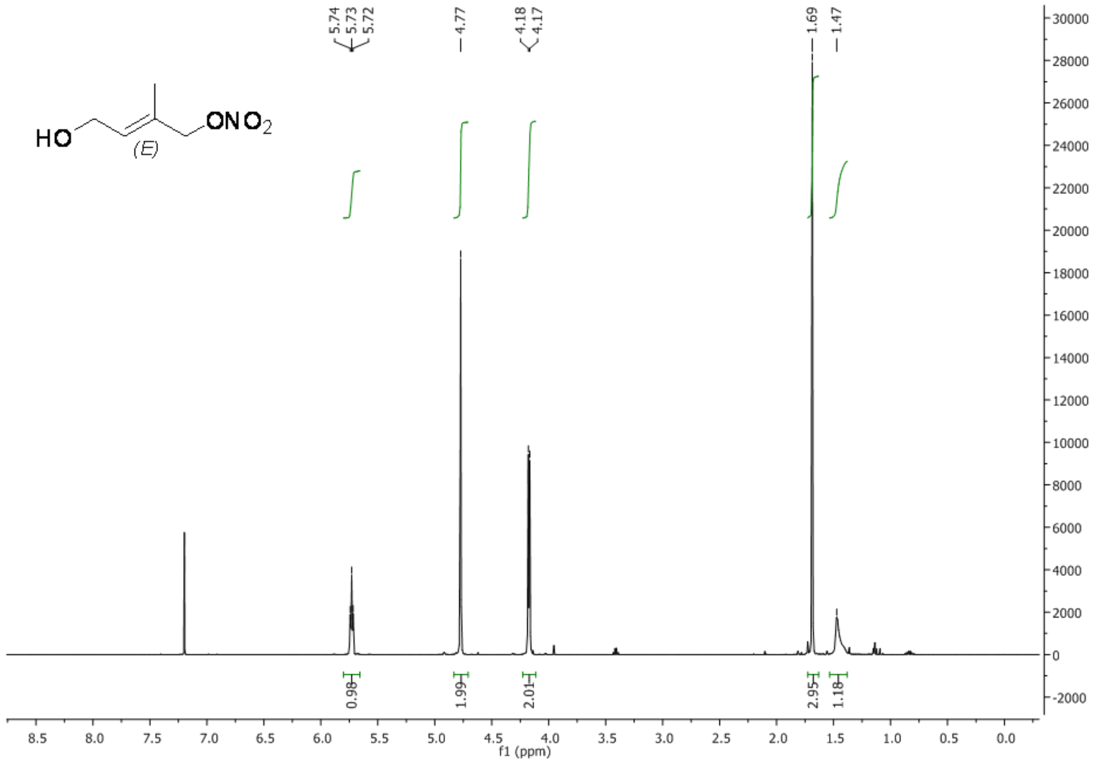

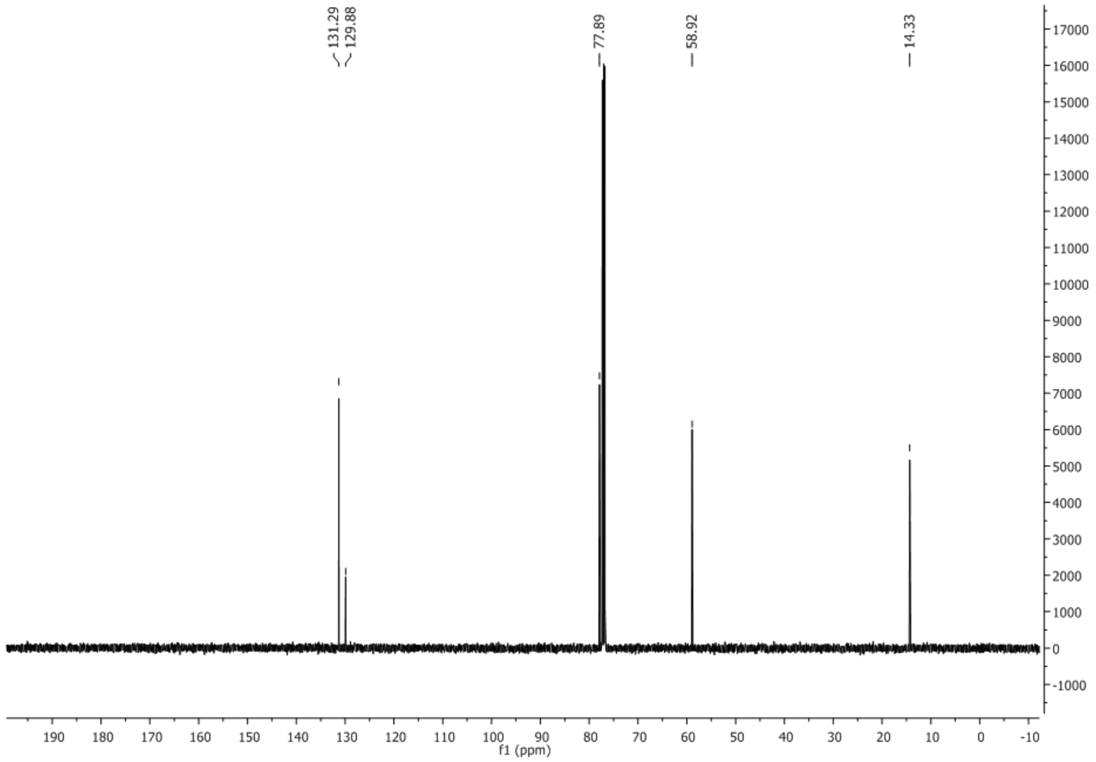

^{1}H NMR and ^{13}C NMR of (*Z*)-**61**

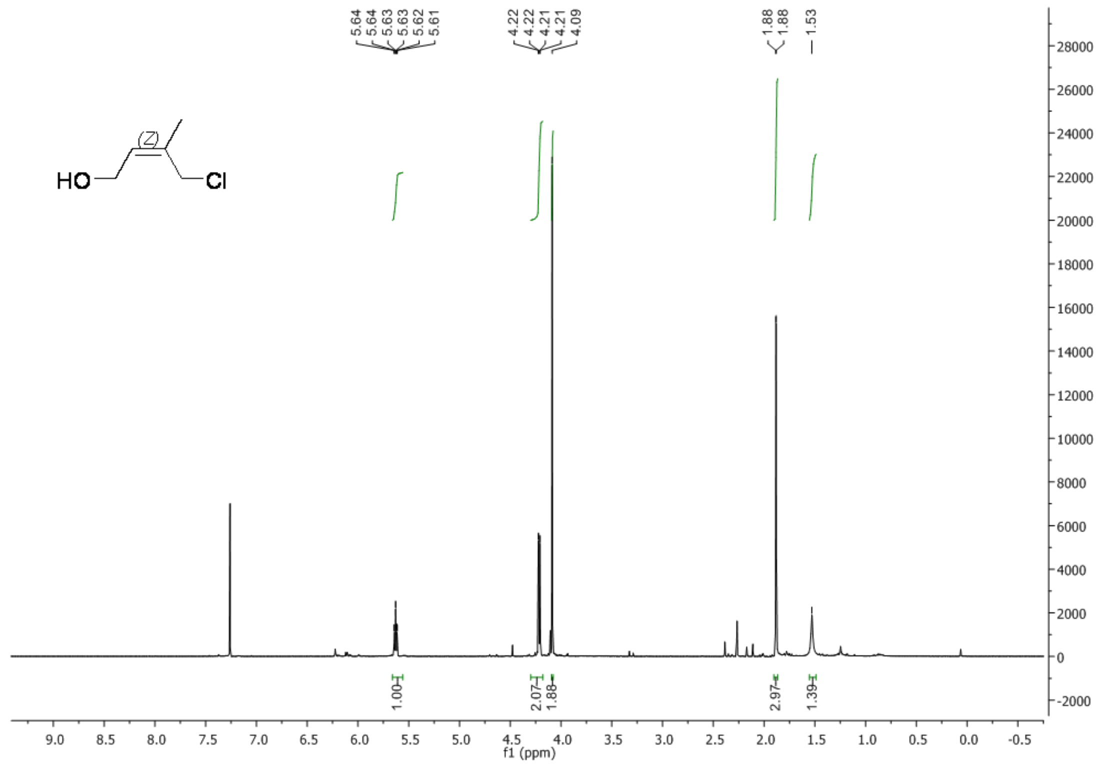

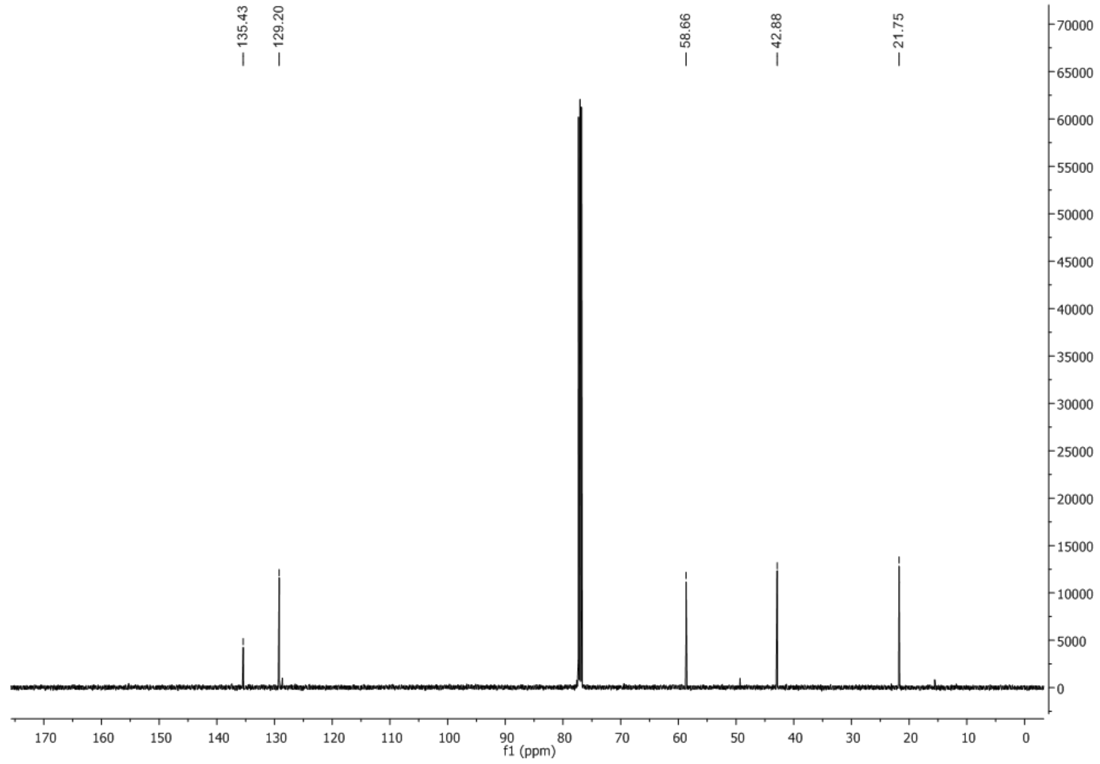

^{1}H NMR and ^{13}C NMR of (Z)-**9**

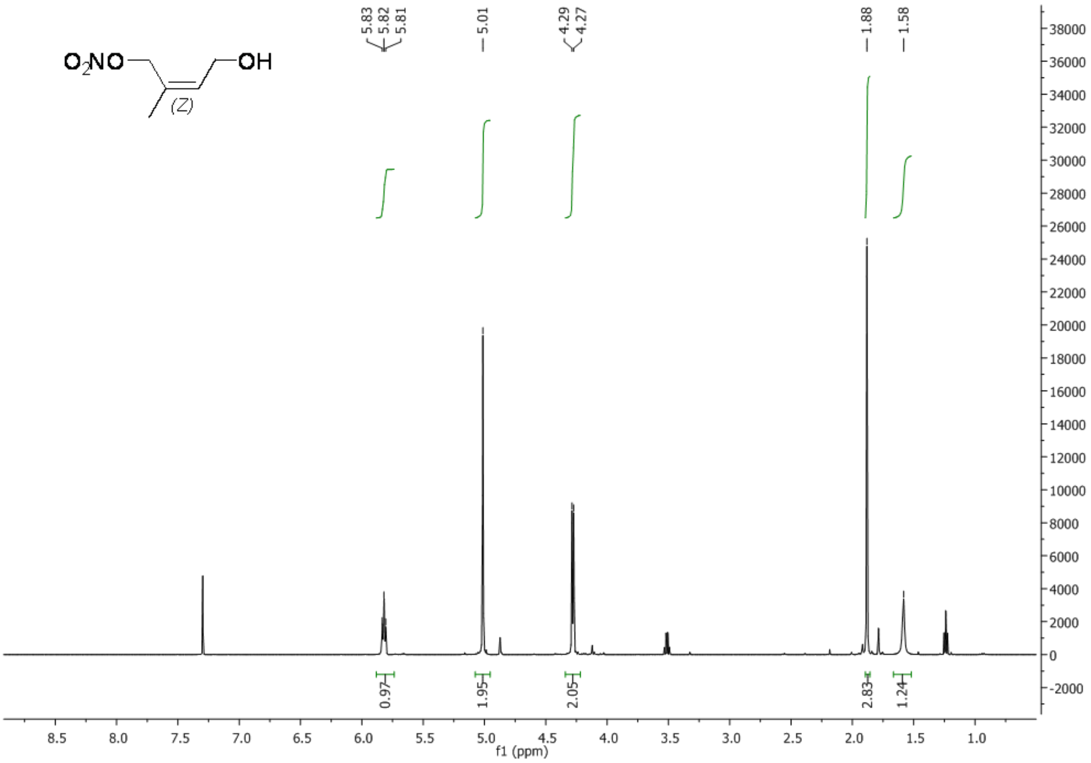

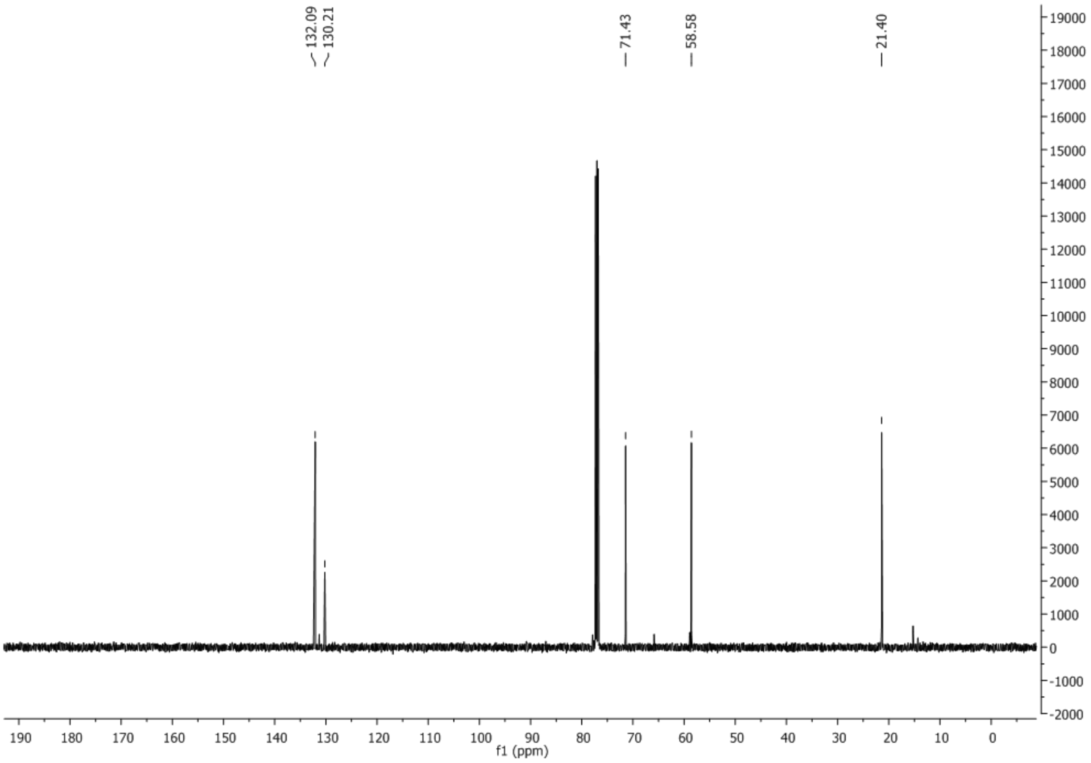

^{1}H NMR and ^{13}C NMR of (E)-**64**

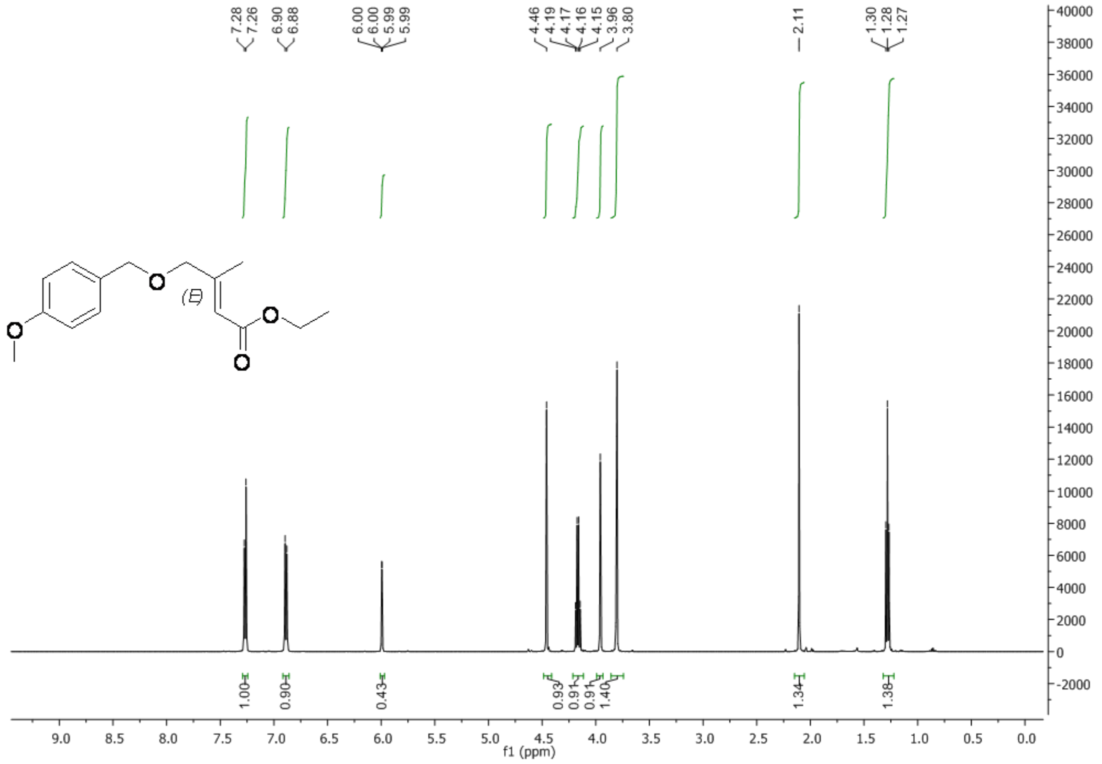

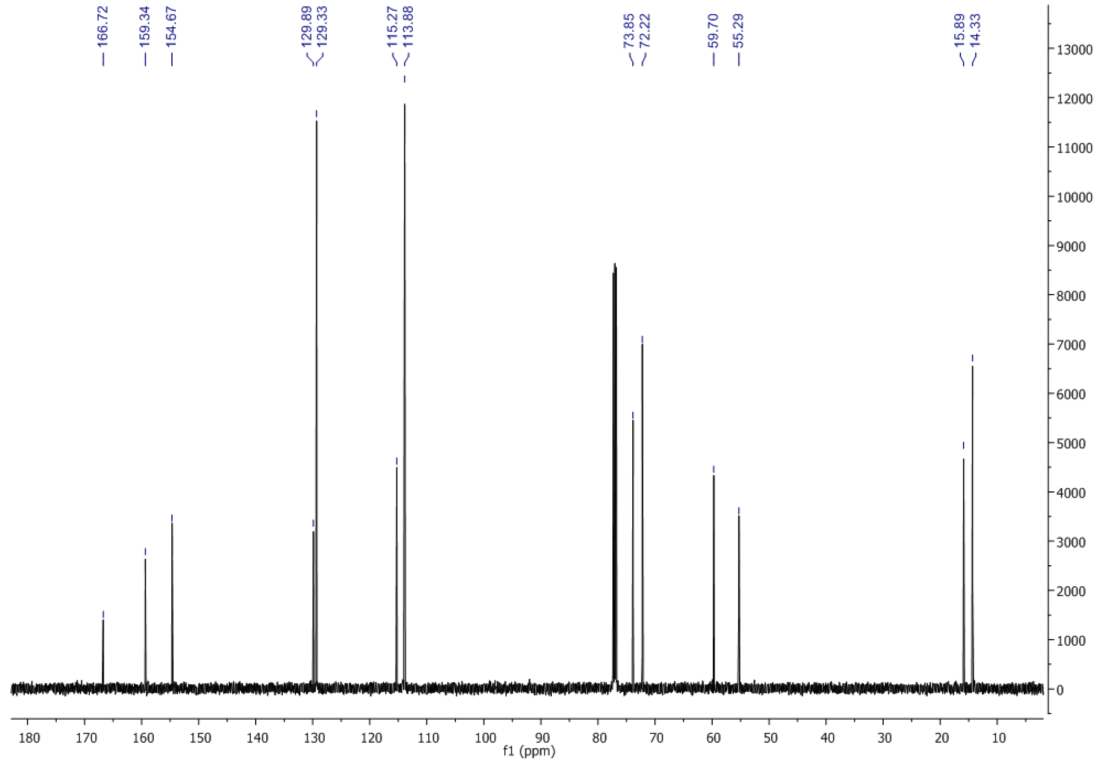

^{1}H NMR and ^{13}C NMR of (Z)-**65**

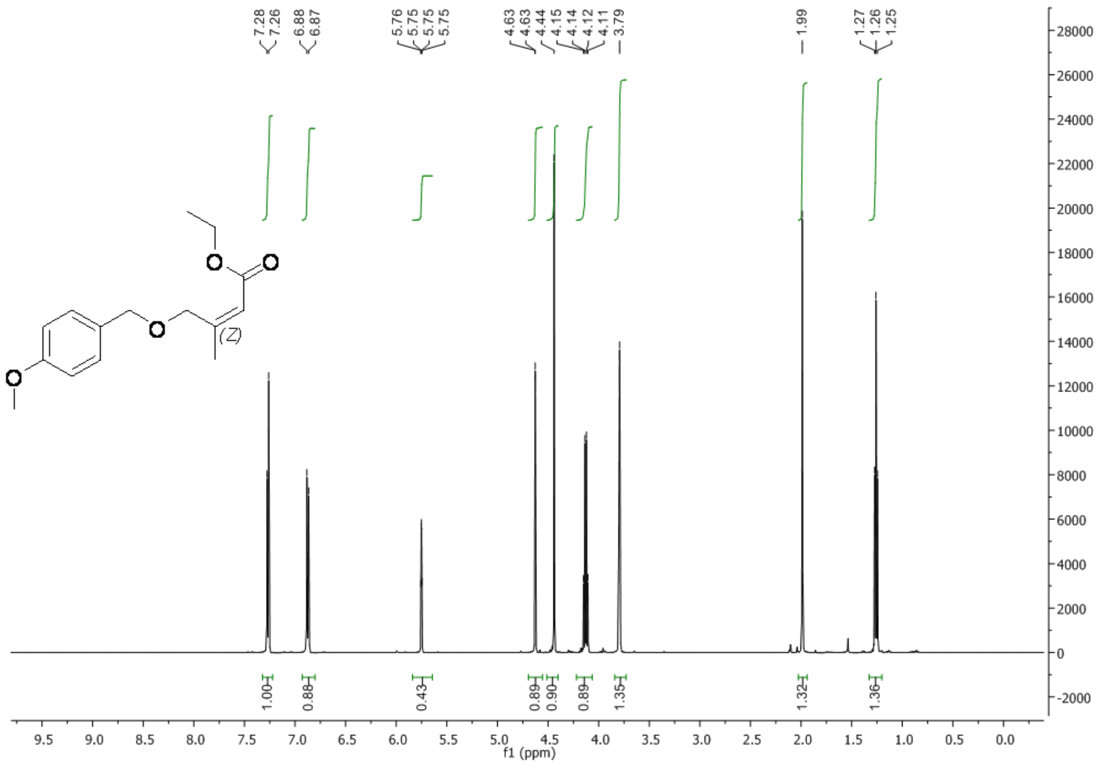

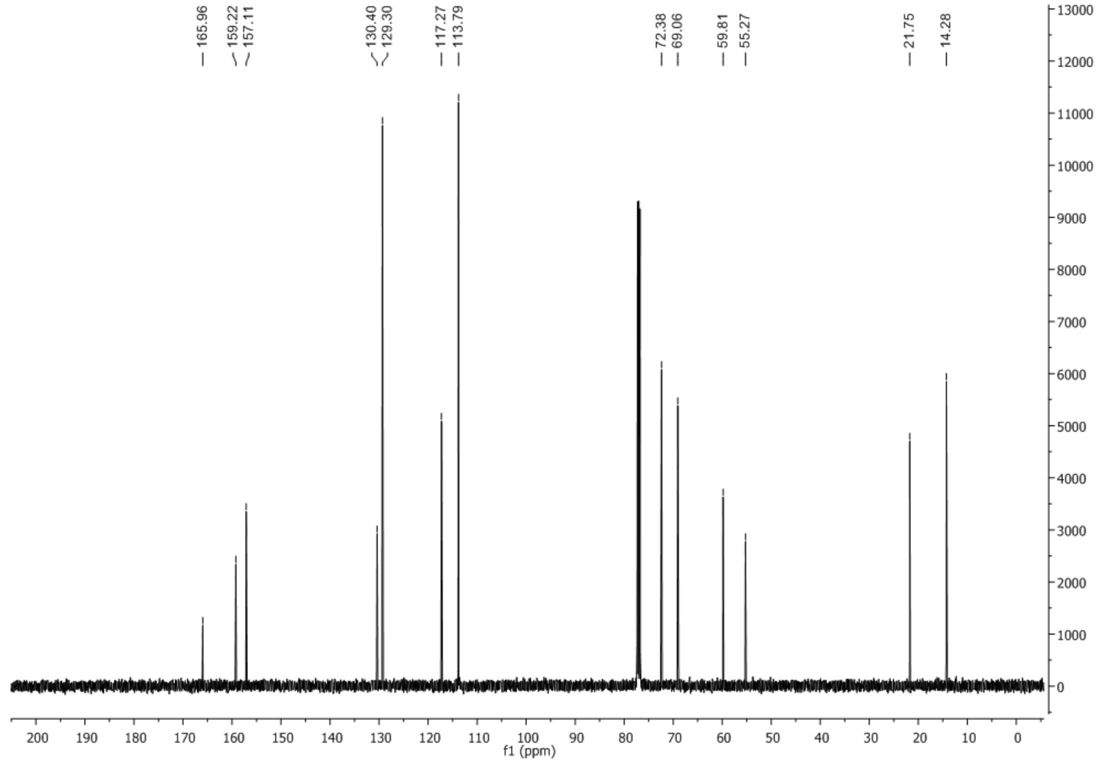

^{1}H NMR and ^{13}C NMR of (E)-**66**

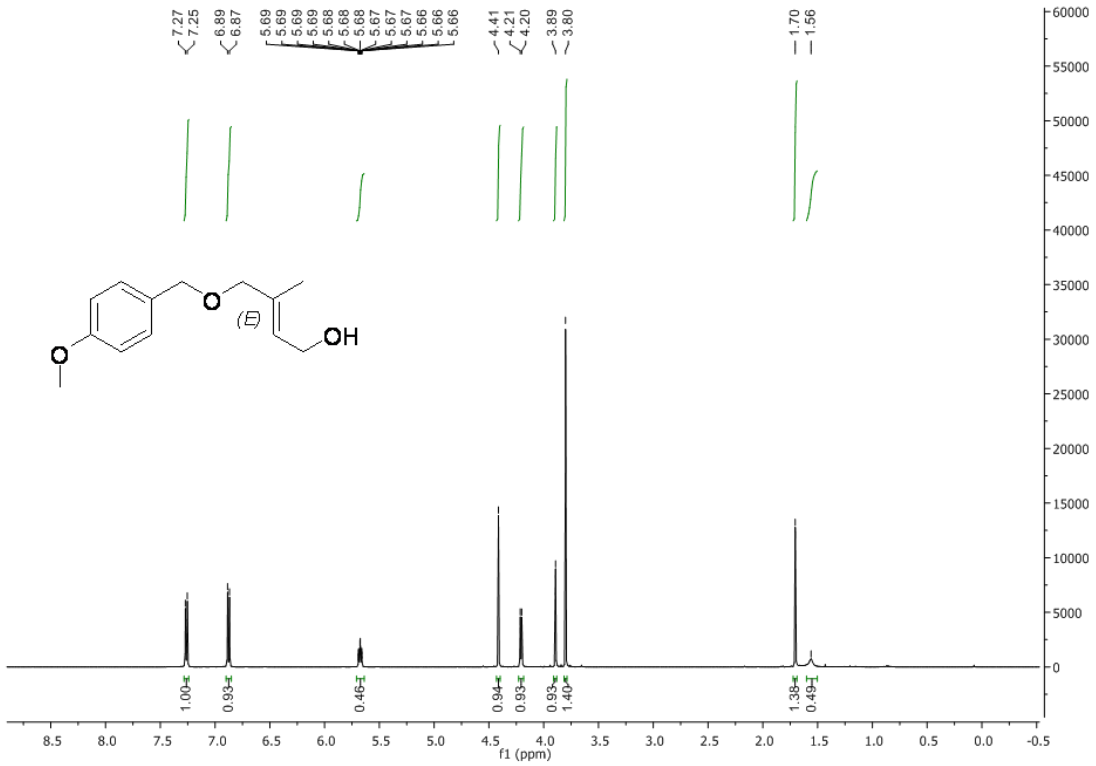

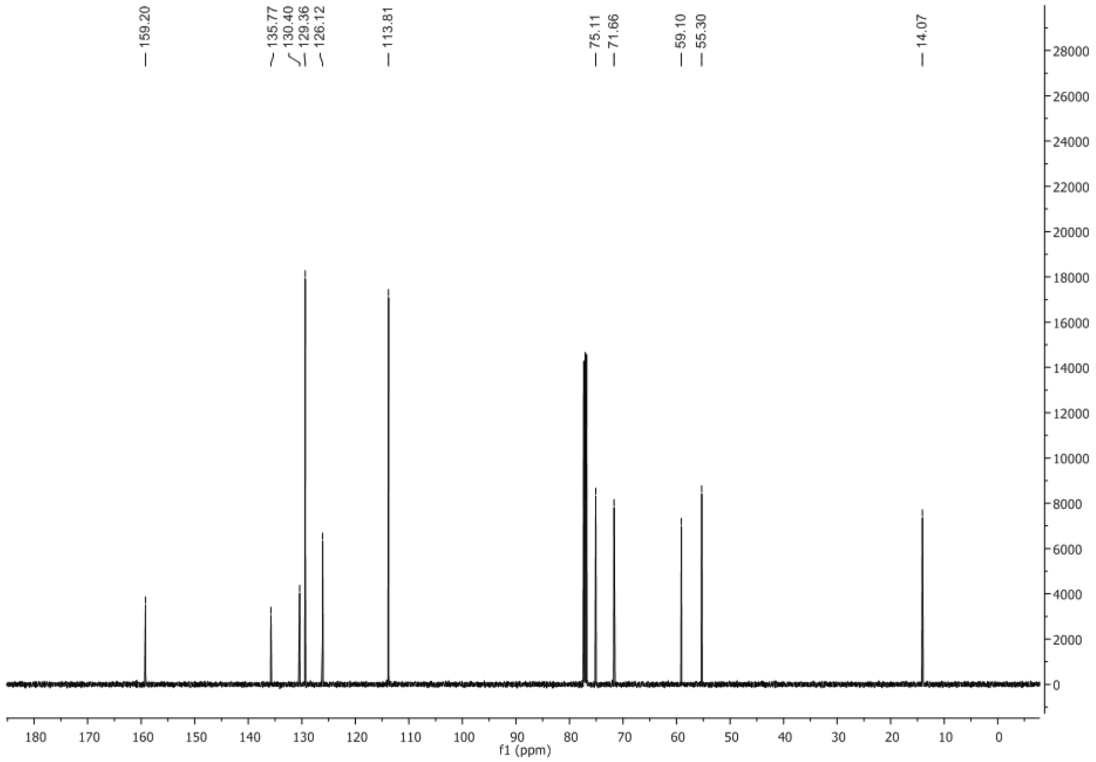

^{1}H NMR and ^{13}C NMR of (E)-**67**

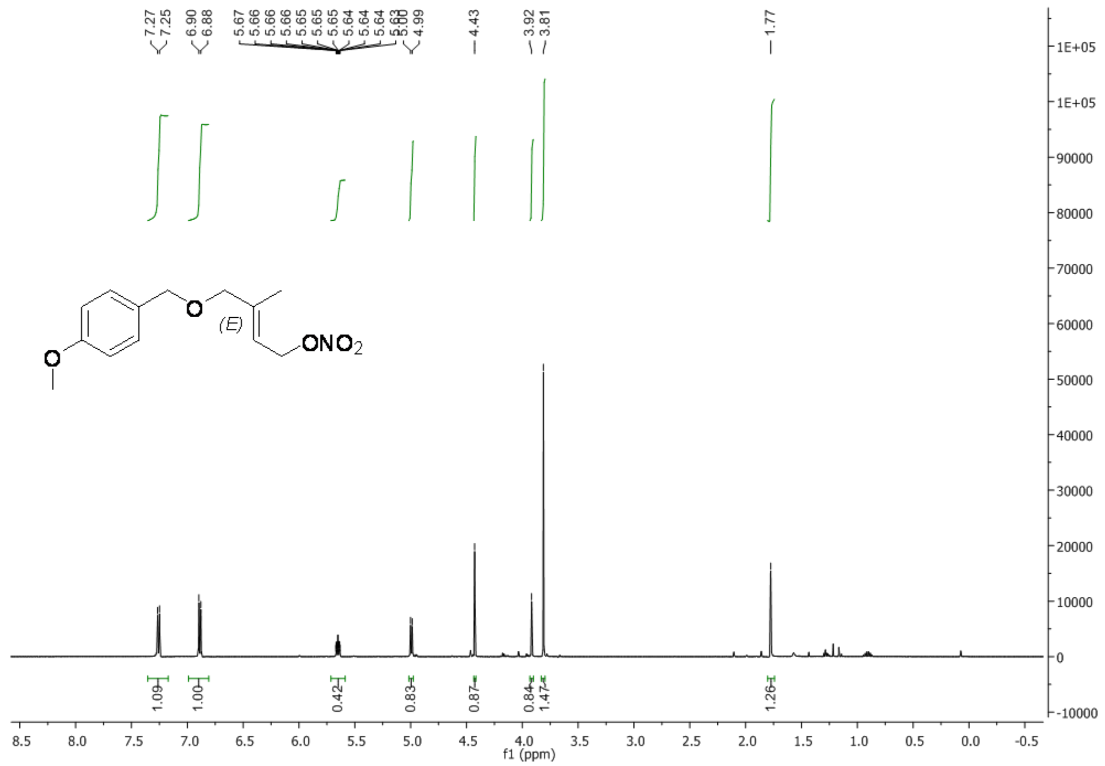

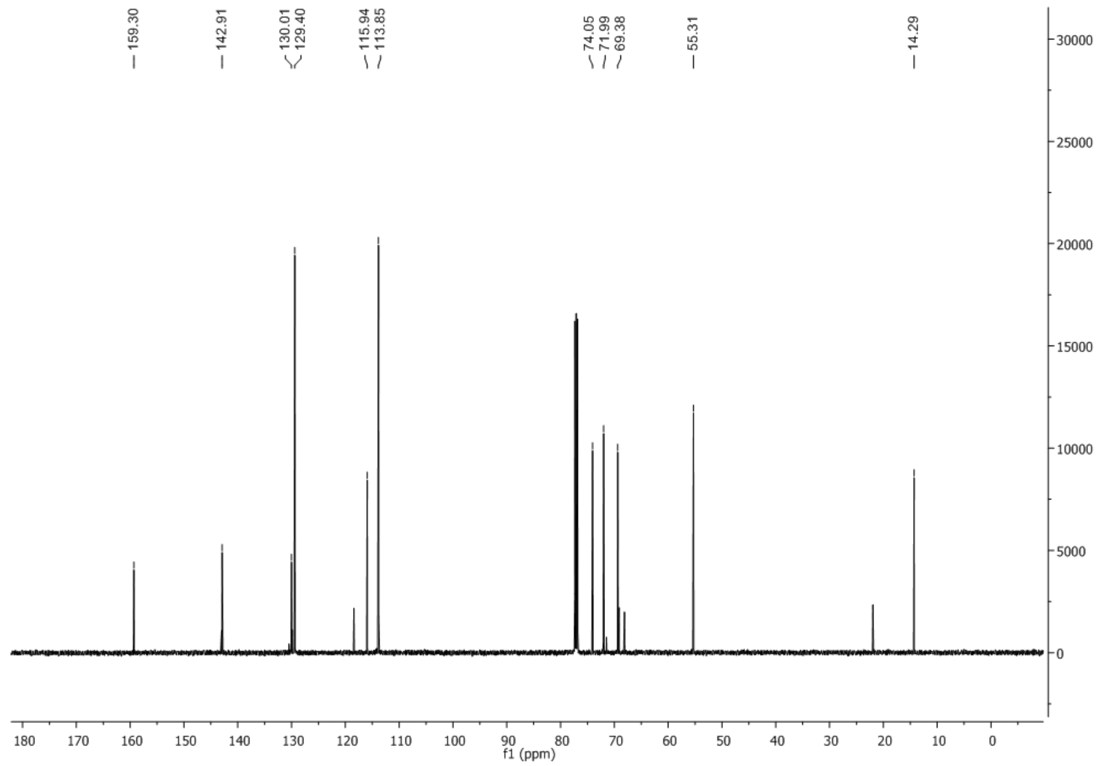

^{1}H NMR and ^{13}C NMR of (*E*)-**11**

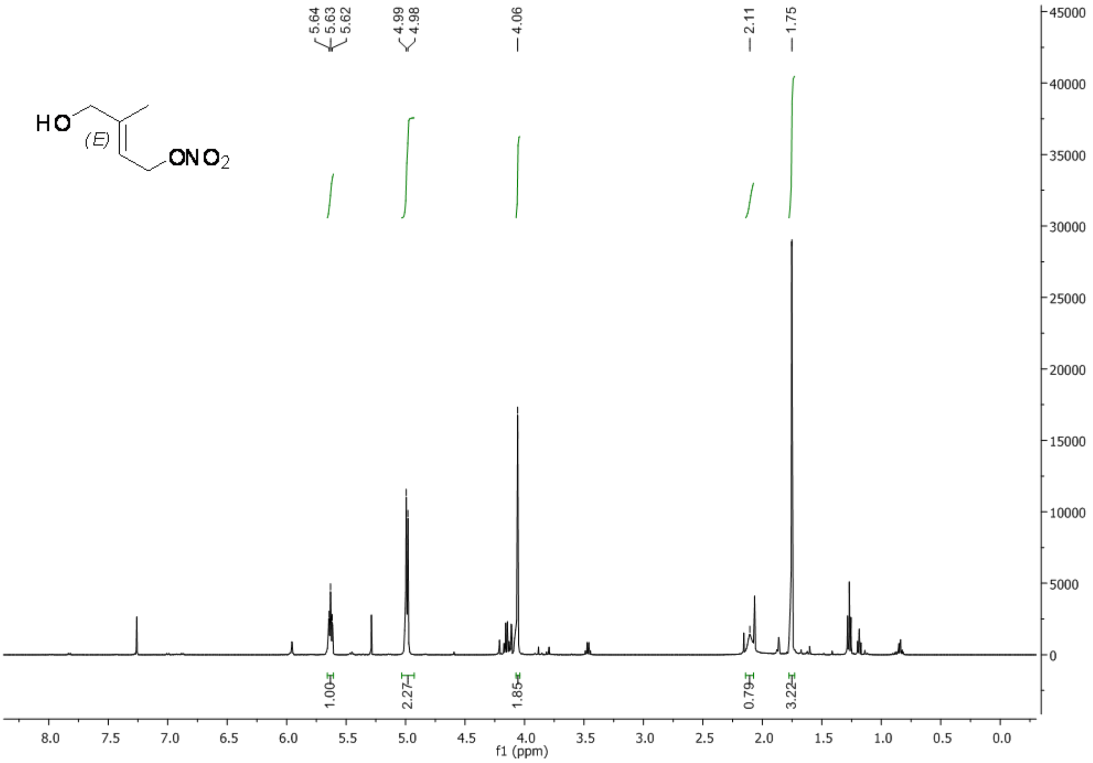

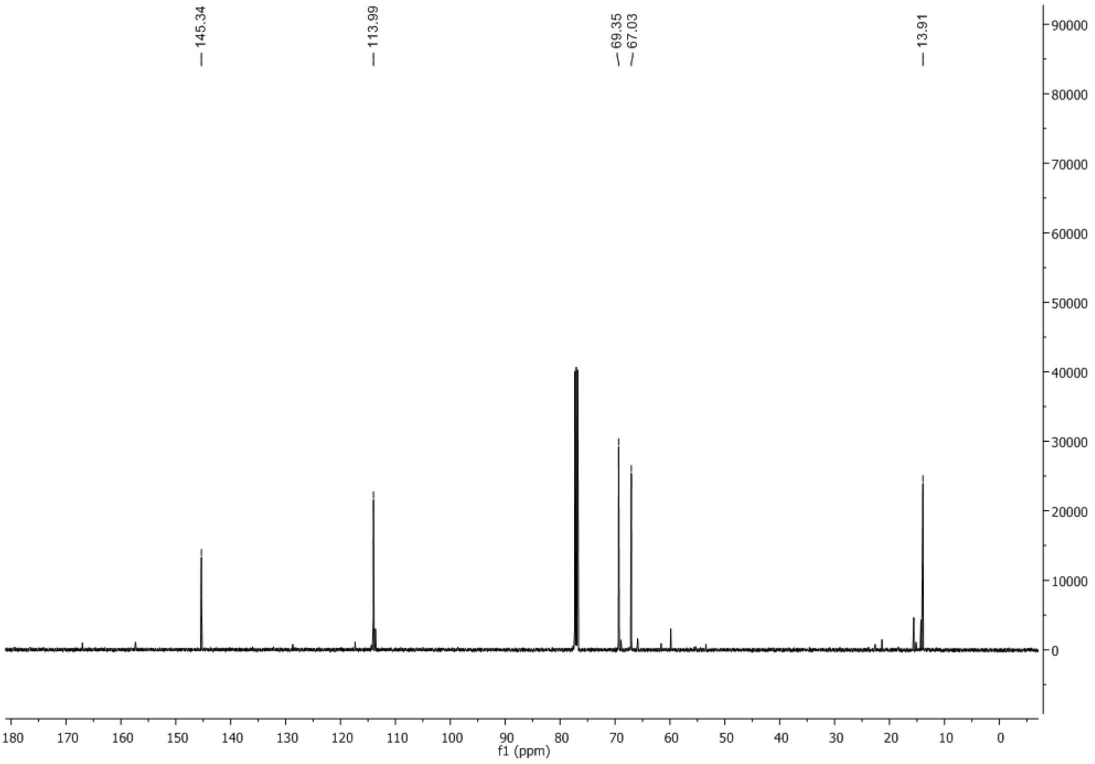

^{1}H NMR and ^{13}C NMR of (*Z*)-**12**

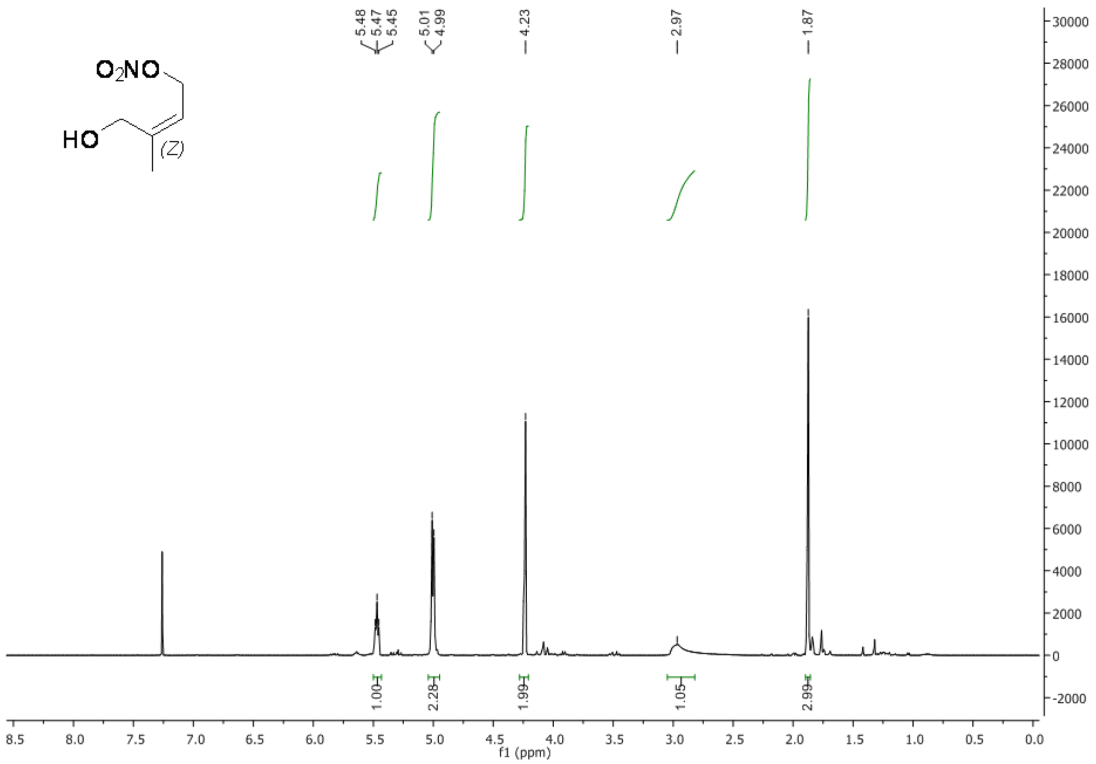

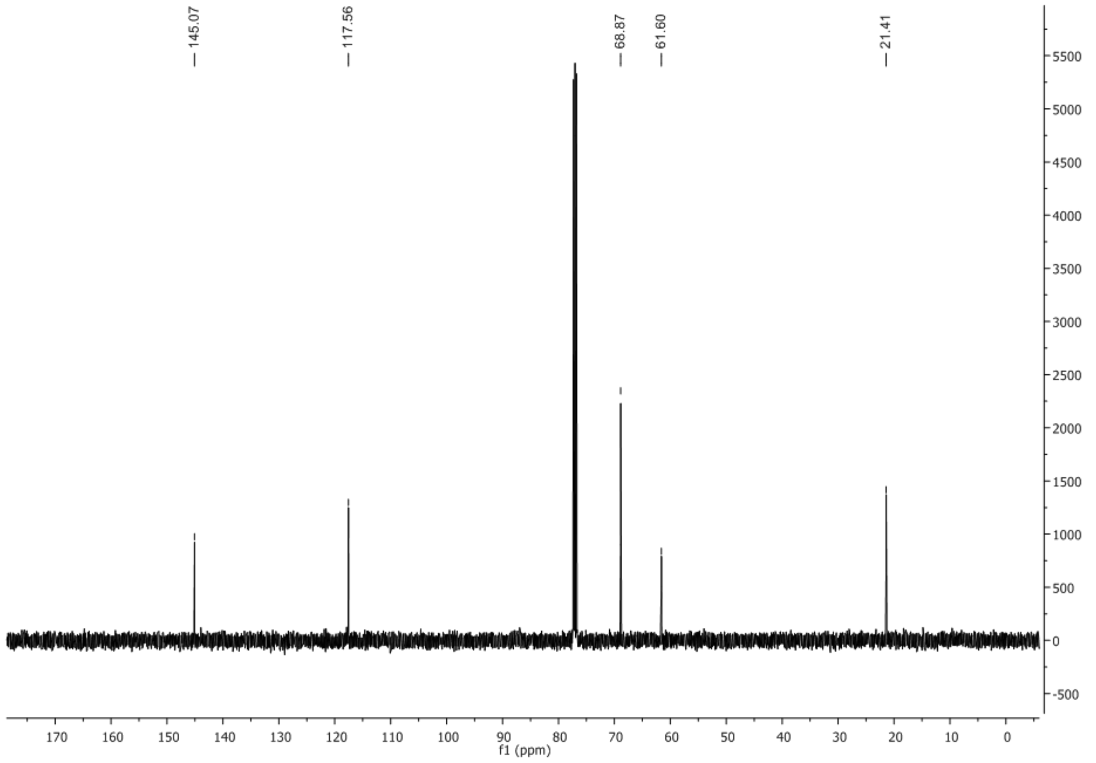

^{1}H NMR and ^{13}C NMR of *rac*-**74**

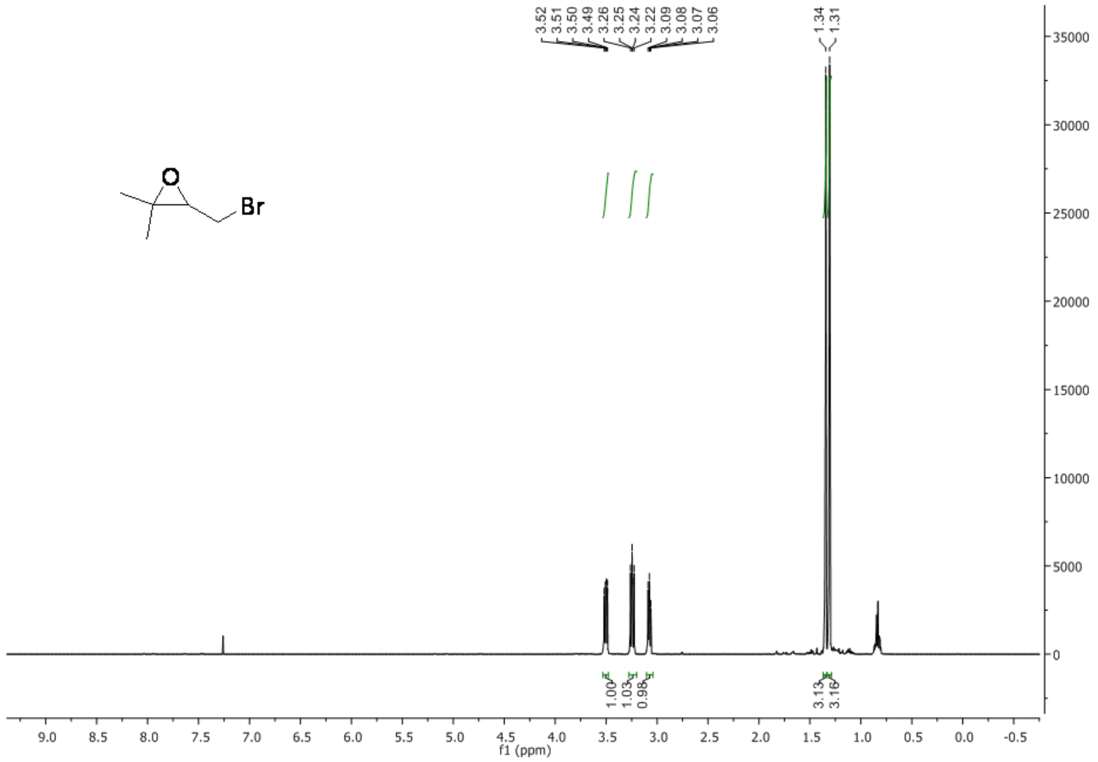

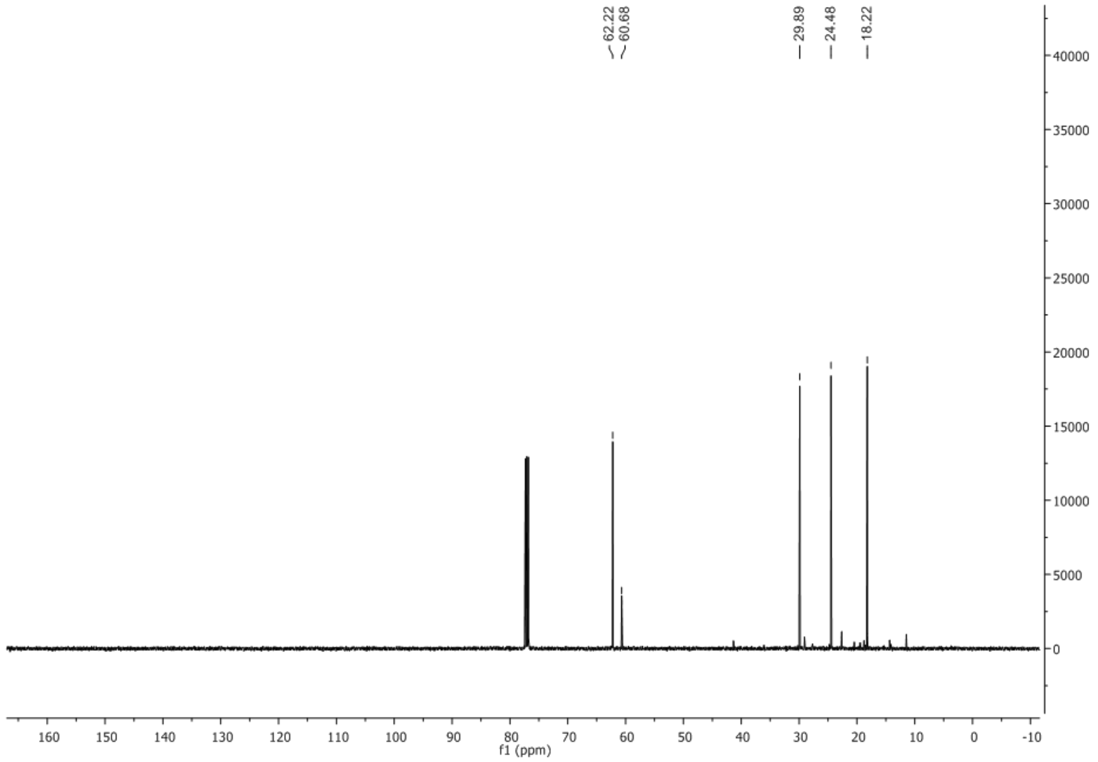

^{1}H NMR and ^{13}C NMR of *rac*-**83**

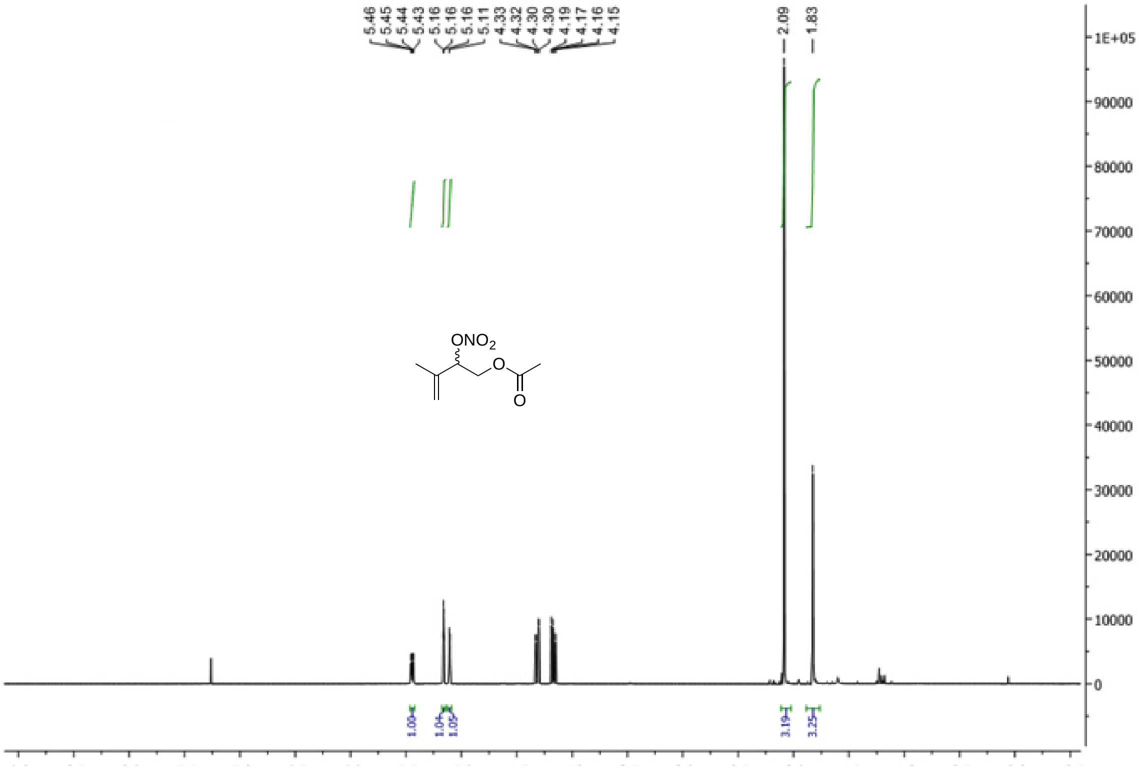

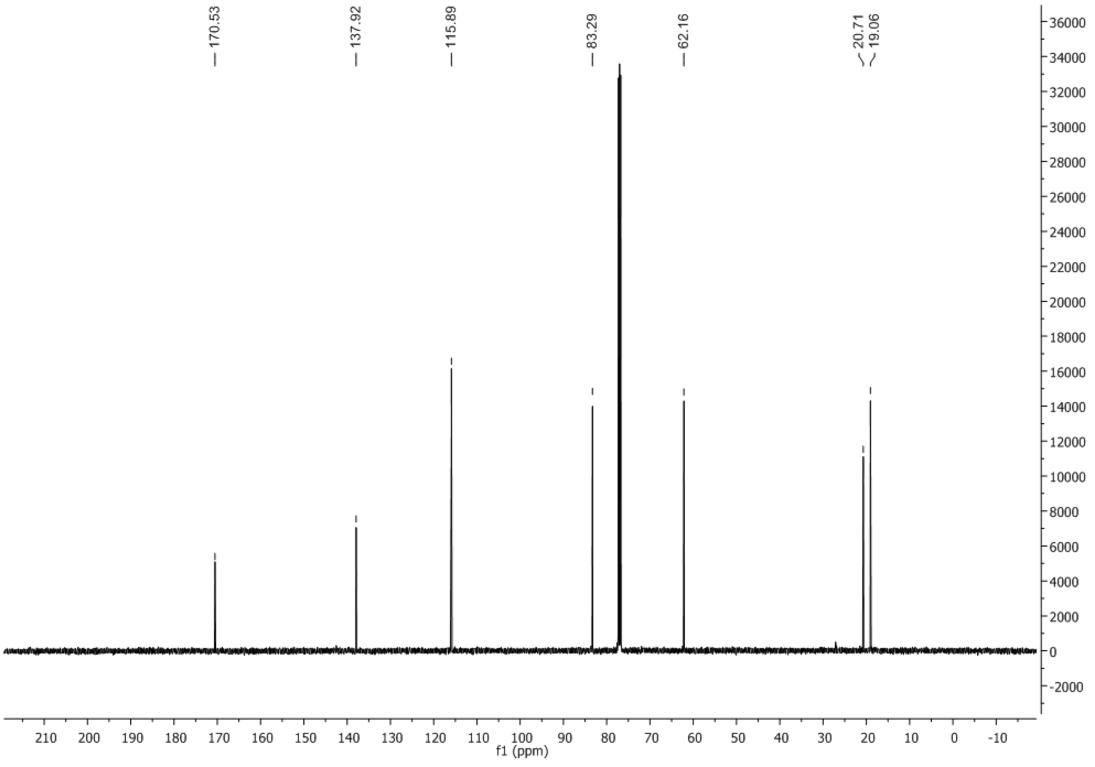

^{1}H NMR and ^{13}C NMR of *rac*-**16**

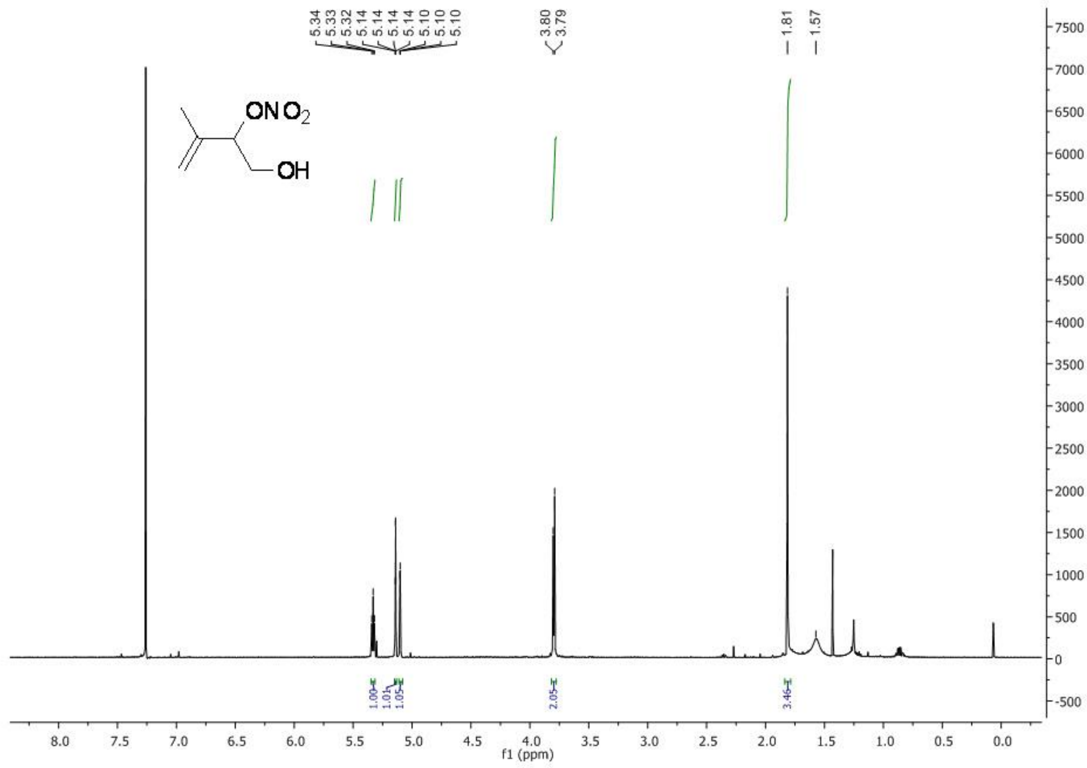

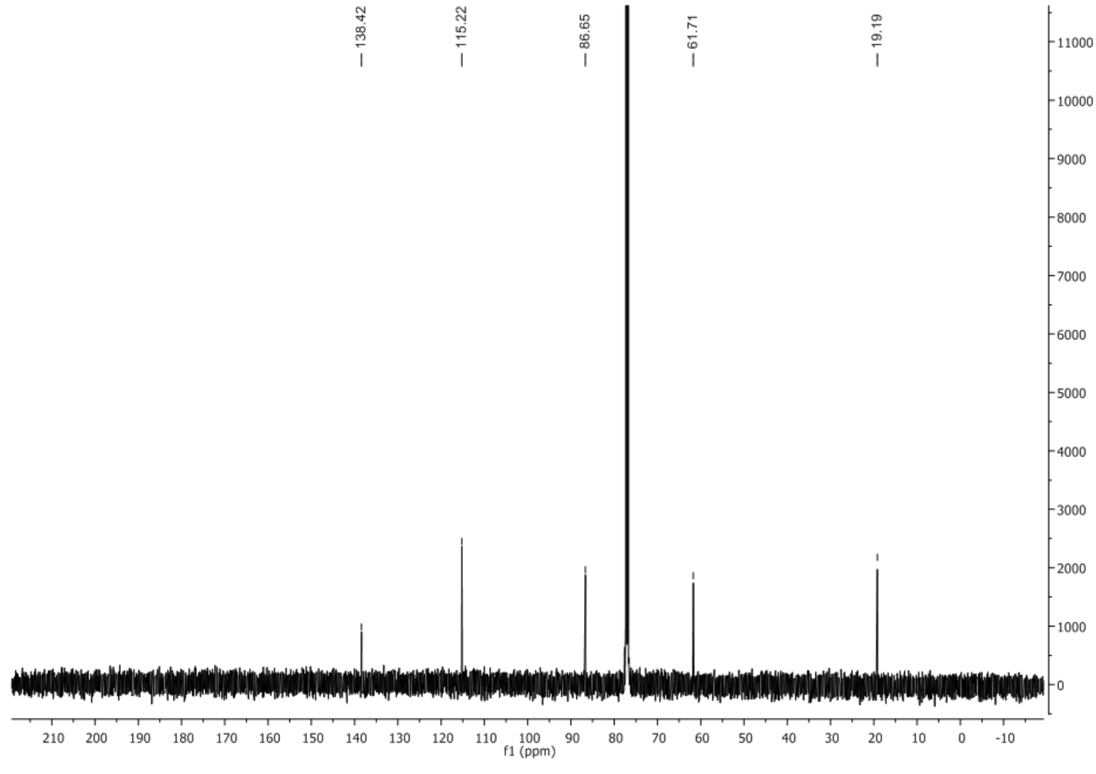

^{1}H NMR and ^{13}C NMR of optically active **86**

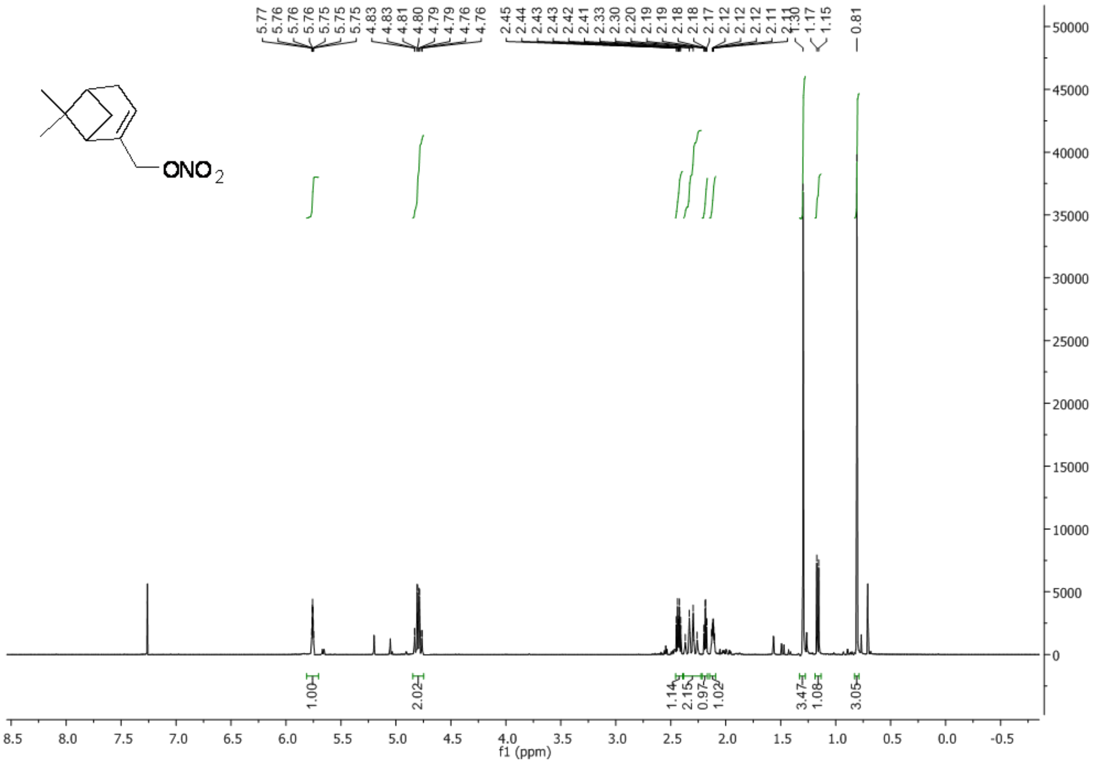

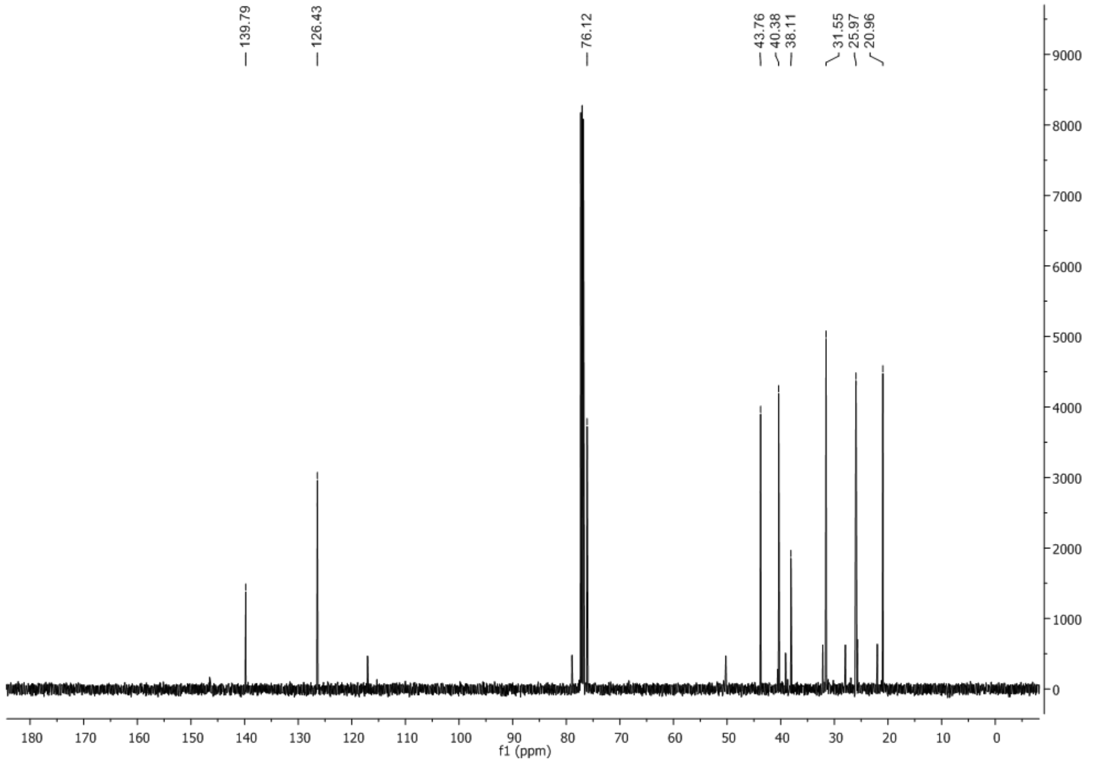

References

[S1] Landis, Y.; Planchenault, D. *Tetrahedron* **1995,** *51*, 12097–12108.

[S2] Ghosh, S. K.; Butler M. S.; Lear, *Tetrahedron Letters*, **2012**, *53*, 2706 - 2708.

[S3] Arnold J. S.; Nguyen, H. M. *J. Am. Chem. Soc.*, **2012**, *134*, 8380 - 8383.

[S4] *Org. Process Res. Dev.*, **2005**, *9*, 278 - 287 doi:10.1021/op049822x

[S5] Gevorkyan, A. A.; Kazaryan, P. I.; Avakyan, S. V.; Simonyan, E. S. *Chemistry of Heterocyclic Compounds*, **1989**, *25*, 254 - 257
